# Water transport in reverse osmosis membranes is governed by pore flow, not a solution-diffusion mechanism

**DOI:** 10.1126/sciadv.adf8488

**Published:** 2023-04-14

**Authors:** Li Wang, Jinlong He, Mohammad Heiranian, Hanqing Fan, Lianfa Song, Ying Li, Menachem Elimelech

**Affiliations:** ^1^ Department of Chemical and Environmental Engineering, Yale University, New Haven, CT 06520-8286, USA.; ^2^ Department of Mechanical Engineering, University of Wisconsin-Madison, Madison, WI 53706-1572, USA.; ^3^ Department of Civil, Environmental, and Construction Engineering, Texas Tech University, Lubbock, TX 79409-1023, USA.

## Abstract

We performed nonequilibrium molecular dynamics (NEMD) simulations and solvent permeation experiments to unravel the mechanism of water transport in reverse osmosis (RO) membranes. The NEMD simulations reveal that water transport is driven by a pressure gradient within the membranes, not by a water concentration gradient, in marked contrast to the classic solution-diffusion model. We further show that water molecules travel as clusters through a network of pores that are transiently connected. Permeation experiments with water and organic solvents using polyamide and cellulose triacetate RO membranes showed that solvent permeance depends on the membrane pore size, kinetic diameter of solvent molecules, and solvent viscosity. This observation is not consistent with the solution-diffusion model, where permeance depends on the solvent solubility. Motivated by these observations, we demonstrate that the solution-friction model, in which transport is driven by a pressure gradient, can describe water and solvent transport in RO membranes.

## Teaser

MD simulations and solvent permeation experiments show that water transport in reverse osmosis membranes is governed by pore flow.

## INTRODUCTION

As the need to augment water supply grows in water-scarce regions of the world, desalination of saline waters such as seawater and brackish groundwater has become crucially important. Reverse osmosis (RO) is the dominant desalination technology, mainly due to its high energy efficiency and low operating costs compared to other desalination technologies (*1*, *2*). RO is also a central component in advanced municipal wastewater reuse, a growing technology for alleviating water scarcity in water-stressed regions (*3*, *4*).

Semipermeable desalination membranes—membranes that allow transport of water and reject salt and other solutes—lie at the heart of the RO technology (*1*, *5*). Because of their outstanding water permeability and water-salt selectivity, thin-film composite (TFC) polyamide membranes have been the gold standard for desalination since their invention more than four decades ago (*6*, *7*). TFC RO membranes are formed by interfacial polymerization of *m*-phenylenediamine and trimesoyl chloride on a porous substrate, resulting in a thin (100 to 150 nm) cross-linked selective layer (*8*, *9*).

The transport of water and salt through the active layer of RO membranes governs the membrane desalination performance. The widely accepted theory or mechanism to describe water and salt transport in RO membranes is the solution-diffusion (SD) model, which was proposed over half a century ago (*10*). This model assumes that the membrane active layer is a “dense” polymer phase, where water molecules “dissolve” into the membrane and then diffuse through the membrane down their concentration gradient (*11*, *12*). Another key assumption in the SD model is that the pressure across the membrane is constant (*13*, *14*). In the absence of a pressure gradient, this assumption implies that the chemical potential gradient of water within the membrane is only expressed as a concentration gradient, which is the driving force for water diffusion through the membrane (*11*, *12*). On the basis of this SD mechanism, water permeance (i.e., permeate water flux normalized by the applied pressure) depends on the “solubility” (partitioning) of water into the membrane and diffusivity of water molecules inside the membrane.

Despite the wide acceptance and use of the SD model, recent findings appear to challenge some of its key assumptions. Advances in electron microscopy revealed the presence of interconnected sub-nanometer cavities and tunnels in fully aromatic polyamide RO membranes (*15*–*19*), in contrast to the assumption in the SD model of dense, nonporous membranes. The average effective pore size or free volume of the polyamide layer of RO membranes has been quantified using positron annihilation lifetime spectroscopy. These studies report a bimodal pore size distribution with peaks around 4 to 5 Å and 7 to 9 Å (*20*, *21*), in general agreement with molecular dynamics (MD) simulations (*22*–*26*). A recent neutron spectroscopy study examined the water dynamics inside a polyamide RO membrane, revealing that water molecules transporting through membrane pores contribute substantially to the overall water flux (*27*). Recent MD simulations further suggest that water molecules transport in chains through connected “pockets” inside the polyamide membrane (*28*, *29*), with a length of continuous water percolation up to 96% of the membrane thickness (*30*). The existence of interconnected pores is also supported by recent experimental studies (*16*, *19*).

Collectively, the experimental and computational studies discussed above may suggest that water molecules transport in chains through interconnected channels inside RO membranes. These studies may therefore imply a pore-flow mechanism for water transport in RO membranes, rather than the widely accepted view of an SD mechanism. Although a recent study suggested that the SD model and the pore-flow model can be mathematically equivalent for swollen polymer membranes (*31*), the two models differ fundamentally in the physical mechanism for water transport in the membrane, thereby shedding no light on the debate about the true mechanism for water transport in RO membranes. Therefore, to address this knowledge gap, there is a critical need to unravel the mechanism and true driving force for water transport in RO membranes, which will be imperative for the development of next-generation desalination membranes.

In this study, we combine MD simulations and solvent permeation experiments to investigate the transport mechanisms of water and organic solvents through RO membranes. Nonequilibrium MD (NEMD) simulations are first used to probe the transport mechanism of water molecules through a polyamide membrane under varying applied pressures. Specifically, we calculate the pressure and concentration of water molecules within the membrane to examine the validity of the key assumptions in the SD model. In addition, trajectories of water molecules in our NEMD simulations are traced to further understand the nature of water transport (i.e., chemical diffusion transport or pressure gradient driven flow). To supplement the computational studies, we performed permeation experiments with water and various organic solvents across two state-of-the-art RO membranes—polyamide and cellulose triacetate—to systematically investigate the role of pressure in solvent permeation. The experimental results with both types of membranes reveal that the solvent permeate flux increases with increasing applied pressure when the solvent molecular size is smaller than the membrane pore size, whereas a critical pressure must be overcome when the solvent molecular size approaches the membrane pore size. Last, we demonstrate that the solution-friction model, where pressure gradient is the driving force, can be used to predict water and solvent transport in RO membranes.

## RESULTS

### Water transport is driven by pressure gradient

To better understand the molecular-level mechanisms of water transport in RO, we performed NEMD simulations of water transport across a polyamide membrane for different applied pressures. The NEMD simulations were carried out using the Large-scale Atomic/Molecular Massively Parallel Simulator (LAMMPS) package (*32*). We describe the methods used to build an atomistic model of the polyamide membrane formed by *m*-phenylenediamine and trimesoyl chloride molecules and summarize the properties of the synthesized membrane in the Supplementary Materials (figs. S1 to S3 and table S1) (*33*–*40*). The computational model for the polyamide membrane has been validated against previous experiments in terms of the atomic composition, density, pore size distribution, water flux, and salt rejection, as described in our recent studies (*25*, *26*).

**Fig. 1.**
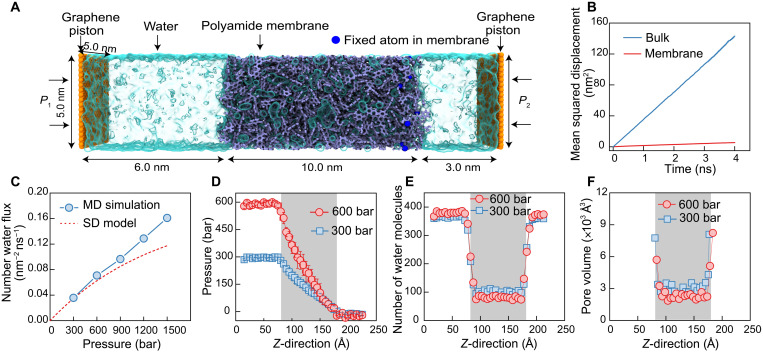
MD simulation of water transport through a polyamide membrane. (**A**) Setup of molecular simulation for water transport through a polyamide membrane. The polyamide membrane (purple) with a thickness of 10 nm is placed between two graphene sheet pistons (orange). The water molecules are visualized as a light blue transparent surface. Hydraulic pressure (*P*_1_) is applied to the left graphene sheet during the simulation, and a standard atmospheric pressure (*P*_2_) is applied on the right graphene sheet, resulting in a pressure difference Δ*P* = *P*_1_ − *P*_2_. (**B**) Mean squared displacement of water molecules inside the hydrated polyamide membrane (red line) compared with bulk diffusion (blue line). (**C**) Water flux through the polyamide membrane as a function of applied pressure. The dashed red line is calculated on the basis of the SD model (details are provided in the Supplementary Materials). (**D**) Pressure distribution, (**E**) number of water molecules, and (**F**) pore volume along *z*-direction through the polyamide membrane under two pressure differences across the membrane (300 and 600 bar).

In Fig. 1A, the simulation box consists of a 10-nm-thick polyamide membrane, water molecules in the feed and permeate reservoirs, and two piston graphene sheets. A driving force is developed by applying a pressure difference (Δ*P* = 300, 600, 900, 1200, and 1500 bar) between the two graphene pistons, where the pressure on the permeate reservoir (*P*_2_) is always maintained at atmospheric pressure (1 bar). For each pressure difference, an NEMD simulation is performed for more than 70 ns. From the mean squared displacement in our equilibrium molecular simulations (Fig. 1B), we calculated the water diffusion coefficient inside the membrane to be 2.48 × 10^−6^ cm^2^ s^−1^, which is one order of magnitude smaller than that of the bulk water. The calculated diffusion coefficient is consistent with the values reported in experiments (*27*), indicating that our atomistic model is a good representation of fully aromatic polyamide membranes.

We note that in our NEMD simulations, we applied pressures that are much higher than those in practical RO operation. Under practical lower pressures, the transport of a single water molecule is considered a rare event within the time scales accessible to NEMD simulations. Therefore, we applied high pressures to accelerate the simulations and collect sufficient statistics for the transport in our 70-ns-long simulations. This is a common strategy used in RO MD simulations (*41*–*43*). In addition, the water flux-pressure curve, shown in Fig. 1C, passes through the origin when linearly extrapolated for lower pressures (i.e., at the origin, the flux and pressure are both zero). This suggests that the water flux is expected to change linearly with pressure for low applied pressures. Because the deviation from linearity based on the SD model occurs for very high pressures (see the dotted curve in Fig. 1C), we are mainly interested in high-pressure transport to assess the validity of the SD model.

Permeate water fluxes at varying applied pressures were calculated by our NEMD simulations (fig. S4). Water flux across the membrane increases linearly with increasing applied pressure (Fig. 1C). This linear relationship contradicts the classic SD model, which predicts a nonlinear dependence at high pressures (*11*, *13*, *31*, *44*). We calculated the permeate water flux based on the SD model using the water diffusion coefficient inside the membrane (Fig. 1B) and the water concentration difference across the membrane (details in the Supplementary Materials) (*11*, *12*, *45*). As seen in Fig. 1C (dashed line), beyond 300 bar, the SD model predicts that water flux deviates from the calculated values in our NEMD simulations. The discrepancy between the simulations and the SD model is more noticeable at higher pressures. When the pressure increases to 1500 bar, the SD model predicts a permeate flux that is ~70% of the flux based on the NEMD simulations. The discrepancy in permeate flux based on the SD model is more severe when the solvent molecules have a larger molar volume. We simulated the permeation of methanol within the same range of applied pressures, revealing a marked deviation from the methanol flux based on the SD model (fig. S5). Our observations from the NEMD simulations can best be described by a viscous-flow model, where a linear relationship exists between solvent flux and applied pressure.

A key assumption of the SD model is that the pressure within the membrane is uniform and equal to the pressure in the feed reservoir; the pressure drops abruptly to the permeate pressure at the interface between the membrane and permeate side (*11*, *12*, *14*). A uniform pressure within the membrane in the SD model implies that the membrane behaves like a liquid when transmitting the pressure through the membrane matrix. To investigate the validity of this assumption, we computed the pressure along the membrane thickness from our NEMD simulations (details in the Supplementary Materials and additional pressure simulations in fig. S6). As shown in Fig. 1D, pressure decreases linearly along the membrane in stark contradiction to the SD model. This observation in our NEMD simulations provides additional evidence in favor of a viscous-flow mechanism driven by a pressure gradient within the membrane.

A consequence of the SD model’s assumption of uniform pressure within the membrane is that the chemical potential gradient is expressed only as a concentration gradient. The concentration gradient of solvent within the membrane according to the SD model is induced by the pressure difference across the membrane. However, the calculated number of water molecules along the membrane from our NEMD simulations show no evidence of a water concentration gradient (Fig. 1E, fig. S7). The absence of a concentration gradient within the membrane clearly indicates that water transport across the membrane cannot be governed by a diffusive mechanism. In Fig. 1E, the number of water molecules inside the membrane is slightly lower at 600 bar compared to 300 bar. This observation is attributed to membrane compaction at high pressures, which slightly reduces membrane porosity (Fig. 1F and figs. S8 and S9). Notably, such slight compaction barely affects the linearity of permeate flux as a function of applied pressure (Fig. 1C), likely due to the increased frequency of connecting adjacent membrane pores or water pockets (*28*, *29*).

### Water molecules permeate through membrane pores in clusters

In the SD model, transport of solvent through the membrane is characterized by the mutual diffusion between the solvent and the membrane (*11*). The Fick’s continuum description of diffusion for such binary systems requires both components to be perfectly mixed such that the mixture is homogeneous in the continuum approximation (*31*). This implies that solvent molecules are well dispersed within the membrane.

**Fig. 2.**
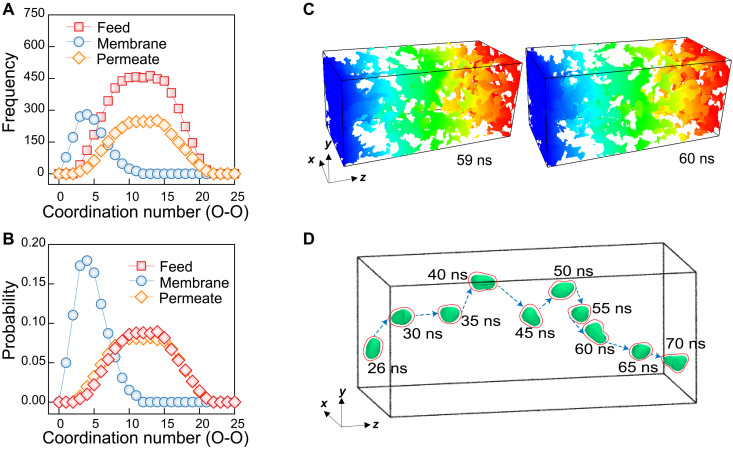
Transport of water clusters in the polyamide membrane. (**A**) Coordination number and (**B**) probability distribution of water molecules in the bulk feed, within the polyamide membrane, and in the permeate after achieving steady state under 300 bar. (**C**) Percolated water-accessible free volume distribution for the polyamide membrane after achieving steady state under 300 bar. Two instantaneous moments at 59 and 60 ns after applying a pressure difference of 300 bar are shown in the figure. The colors denote the depth in the z direction: Blue represents the interface between the feed and the membrane, and red represents the interface between the permeate and the membrane. (**D**) Trajectory of a water cluster containing five water molecules transporting in the membrane under pressure gradient over a simulation duration of 70 ns.

To understand the structure and aggregation of water molecules within the polyamide membrane, we calculated the coordination number of water molecules in the bulk reservoirs (feed and permeate) and within the membrane from our NEMD simulations (Fig. 2, A and B and fig. S10). In these calculations, the total number of water molecules that a single water molecule holds as its nearest neighbor—within a cutoff distance of 0.5 nm—is counted. As shown in Fig. 2, A and B, most water molecules inside the polyamide membrane are surrounded by approximately four other water molecules. As water molecules are confined in the pores of the membrane, their coordination number within the membrane is expected to be lower than the number in the bulk reservoirs. Contrary to the SD model that assumes randomly dispersed water molecules diffusing through the membrane, the nonzero coordination number in the simulations suggests that water molecules are cohesively connected through networks of pores inside the membrane. As illustrated in Fig. 2C, these pores are not permanent, but rather, they are continuously changing by the thermal motion of the polymer matrix, consistent with previous MD simulations (*46*).

To gain deeper insights into the structure of water molecules as they travel through the membrane under a pressure difference, we tagged five water molecules when they entered the membrane on the feed side as shown in Fig. 2D. The trajectory of these tagged water molecules is monitored throughout their complete transport to the permeate side. These tagged water molecules transport together as a cluster (i.e., without being dispersed) through a series of pores inside the membrane that are transiently connected. Figure 2D demonstrates a complete passage for the tagged water molecules from the feed to the permeate end of the membrane. The presence of a feed-to-permeate passage that is long enough for the time scales of water flow through the membrane strongly suggests that a pore-flow mechanism governs water transport in the polyamide membrane.

### Solvent transport is governed by molecular size, not solvent solubility

We further performed systematic permeation experiments of water and organic solvents through the polyamide and cellulose triacetate RO membranes. The solvents tested include water, methanol, ethanol, formamide, isopropanol, *n*-propanol, 2-butanol, and *n*-butanol. The relevant properties of the solvents are provided in Table 1. Among these solvents, we found that 2-butanol and *n*-butanol can permeate through the cellulose triacetate membrane but not through the polyamide membrane. The kinetic diameters of 2-butanol and *n*-butanol (Table 1) are larger than the reported average pore size of polyamide membrane (i.e., ~0.5 nm) (*8*), indicating that size (steric) effects are important in determining solvent permeance.

**Table 1.** Hansen solubility and physical properties of tested solvents. PA, polyamide; CTA, cellulose triacetate membranes.

Solvent	Hansen solubility parameter (MPa^1/2^)	Kinetic diameter *d*_0_ (nm)	Viscosity (mPa‧s)	Permeable (yes/no)	
				PA	CTA
Water	47.8	0.26	0.89	Y	Y
Methanol	29.7	0.36	0.54	Y	Y
Ethanol	26.6	0.45	1.20	Y	Y
Formamide	36.7	0.45	3.23	Y	Y
Isopropanol	24.6	0.469	1.96	Y	Y
*n*-Propanol	23.6	0.47	2.05	Y	Y
2-Butanol	20.8	0.504	3.13	N	Y
*n*-Butanol	23.1	0.505	2.52	N	Y

**Fig. 3.**
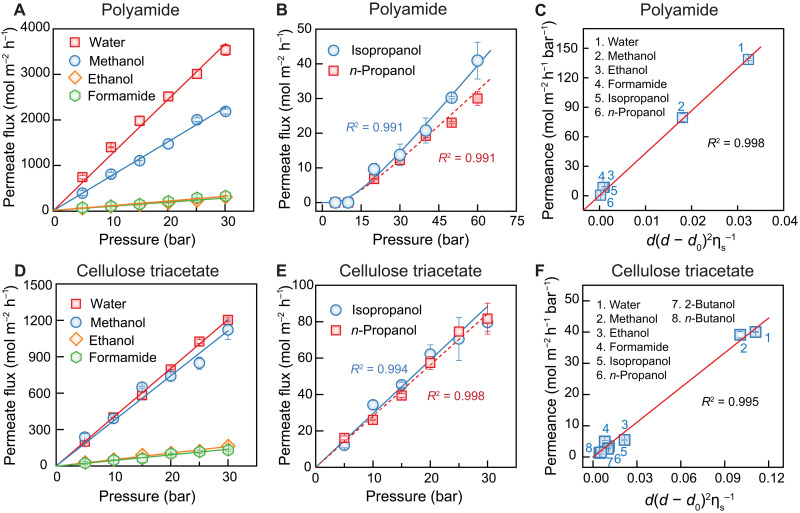
Experimental results of water and organic solvent transport through polyamide and cellulose triacetate RO membranes. (**A**) Linear and (**B**) nonlinear dependence of solvent permeate flux on applied pressure in polyamide RO membranes. The lines in (A) are drawn on the basis of a linear regression, while the curves in (B) are plotted on the basis of Eq. 1. (**C**) Solvent permeance of polyamide RO membranes as a function of a model parameter combining membrane pore size (*d*), solvent size (*d*_0_), and solvent viscosity (η_s_). Note that the solid line is fitted to Eq. 2. The reported permeances of water, methanol, ethanol, and formamide were measured at 10 bar, while the permeances of isopropanol and *n*-propanol were measured at 60 bar. (**D** and **E**) Linear dependence of solvent permeate flux on applied pressure in cellulose triacetate membranes. (**F**) Solvent permeance of cellulose triacetate membranes as a function of a model parameter combining membrane pore size, solvent size, and solvent viscosity. The reported permeances of all solvents were measured at 10 bar. Note that the solid line is fitted to Eq. 2. The open symbols are experimental data, and error bars represent one standard deviation from duplicate experiments.

For solvents that have a relatively small kinetic diameter (i.e., ≤0.45 nm), their permeate fluxes increase linearly with applied pressure (Fig. 3, A and D), with *R*^2^ > 0.99 for each solvent (table S2). The linear dependence of permeate flux on hydraulic pressure contradicts previously reported permeation experiments, where the flux starts to deviate from linearity at high pressures, approaching “ceiling fluxes” for a variety of organic solvents permeating through swollen polymer films (*13*, *44*). As the membrane changes from polyamide to cellulose triacetate, the permeate flux of each solvent decreases markedly because of the increased thickness of the selective layer of the asymmetric cellulose triacetate membrane (the entire cellulose triacetate layer is ~50 μm) compared to the polyamide active layer (~150 nm). For both types of membranes, the permeate flux decreases as the solvent kinetic diameter increases. Specifically, water permeates through the membranes at the fastest flux, followed by methanol, ethanol, and formamide. The permeate flux of ethanol is not distinguishable from that of formamide, because they have the same kinetic diameters.

We observed notably different behaviors for the permeation of isopropanol and *n*-propanol through the polyamide membranes compared to the other solvents. In contrast to the relatively small molecules shown in Fig. 3A, no permeate volume could be measured for isopropanol and *n*-propanol until the pressure increased beyond 20 bar (Fig. 3B). In addition, the permeance for isopropanol and *n*-propanol was found to depend on the applied pressure (fig. S11), indicating a nonlinear dependence of permeate flux on pressure. A recent theoretical study revealed that a chain of solvents must overcome a critical energy barrier before being able to permeate through the membrane pores (*47*, *48*), resulting in a nonlinear dependence of permeate flux on applied pressure (see derivation in the Supplementary Materials) (*49*–*53*)

$$J=k\left(\frac{P}{P_{c}}\right)\exp\left(-\frac{P_{c}}{P}\right)$$ (1)

where *J* is the permeate flux, *k* is a proportionality factor, *P* is the applied pressure, and *P*_c_ is the critical pressure. On the basis of Eq. 1, *J* increases nonlinearly with pressure when *P* is small but approaches a linear regime as *P* outweighs *P*_c_.

As shown in Fig. 3B, Eq. 1 fits the relationship between permeate flux and applied pressure for iso- and *n*-propanol exceedingly well (*R*^2^ = 0.991). Such nonlinear behavior is not observed for the other solvents tested because of their relatively small molecular size. A physical model (eq. S20) demonstrates that *P*_c_ is inversely proportional to the difference between the pore size (*d*) and the kinetic diameter of solvent molecules (*d*_0_). Thus, for relatively small molecules, such as water, methanol, ethanol, and formamide, *P*_c_ is very small and can be easily overcome, leading to a linear dependence regardless of the magnitude of the applied pressure. In contrast, the kinetic diameters of iso- and *n*-propanol are close to the pore size of the polyamide active layer, resulting in an appreciable influence of *P*_c_. The calculated critical pressures for iso- and *n*-propanol (by fitting to Eq. 1) were 12.9 and 13.3 bar, respectively (table S3). The magnitudes of *P*_c_ for the iso- and *n*-propanol are comparable because of their similar kinetic diameters (Table 1).

The dependence of *P*_c_ on the solvent molecular size and membrane pore size is further verified by permeating iso- and *n*-propanol through cellulose triacetate membranes—a membrane with a larger pore size than polyamide membranes [0.65 nm (*54*, *55*) versus 0.5 nm (*8*), respectively]. Figure 3E shows that *P*_c_ for iso- and *n*-propanol as they permeate through cellulose triacetate membranes is nonobservable, resulting a linear increase in the permeate flux as the pressure increases. Because of the similar kinetic diameters of iso- and *n*-propanol, their permeances (i.e., slopes of the curves) are nearly identical.

By extending the physical model introduced previously, we find that the solvent permeance (*A*) is related to the membrane pore size (*d*), kinetic diameter of the solvent molecules (*d*_0_), and solvent viscosity (η_s_) via the following equation (details in the Supplementary Materials)

$$A\propto \frac{d{\left(d-d_{0}\right)}^{2}}{\eta_{s}}$$ (2)

Equation 2 underscores the critical role of membrane pore size in governing solvent permeance, in marked contrast to the SD mechanism. We note that a recent study on organic solvent transport in polyamide nanofilms proposed that the solvent permeance is correlated to the solvent Hansen solubility parameter (eq. S22), within the framework of the SD mechanism (*56*). However, such correlation fails to explain the solvent permeance data in our study, particularly through the cellulose triacetate membranes (fig. S12). Solvents with similar Hansen parameters permeate through the membranes at drastically different rates (table S4), further indicating the inadequacy of solvent solubility in determining solvent permeability. Conversely, Eq. 2 predicts the solvent permeance in polyamide and cellulose triacetate membranes exceptionally well (Fig. 3, C and F), where *d* is set as 0.50 (*8*) and 0.65 nm (*54*, *55*) for the polyamide and cellulose triacetate membranes, respectively. Because the permeances of iso- and *n*-propanol in polyamide membranes depend on pressure, they were taken as those measured at 60 bar (Fig. 3C), while for the other solvents the permeances were measured at 10 bar. Collectively, our results emphasize the significance of membrane pore size and solvent molecular size, rather than solvent solubility, in governing solvent permeance in RO membranes.

### Water and solvent transport in RO membranes can be described by the solution-friction model

As discussed in the previous sections, water transport through RO membranes is governed by a pore-flow mechanism where the pressure linearly decreases within the membrane because of the frictional forces acting on water molecules. The solution-friction (SF) model is derived from a balance of forces acting on the water molecules and salt ions inside the membrane (*57*–*60*). As water travels through the membrane, the hydrostatic pressure is balanced by the friction between the water molecules and pore walls as well as the friction between salt ions and water molecules. The force balance is expressed as (*57*–*59*).

$$\frac{dP^{t}}{dx}=-RTf_{f-m}v_{f}+RT\sum_{i}f_{i-f}c_{i}(v_{i}-v_{f})$$ (3)

Here, *P^t^* is the total pressure (i.e., the applied hydrostatic pressure minus the osmotic pressure), *x* is distance along the membrane thickness, *R* is the gas constant, *T* is the absolute temperature, *c_i_* is the concentration of ion *i* inside the membrane, *f*_*f*−*m*_ and *f*_*i*−*f*_ are the friction coefficients between the water and membrane and between ion *i* and water, respectively, and *v_f_* and *v_i_* are the water velocity and ion *i* velocity inside the membrane, respectively. Equation 3 indicates that the permeate flux, which is proportional to *v_f_*, is coupled with the salt transport (i.e., *c_i_* and *v_i_*). Detailed derivation of the SF model is provided in the Supplementary Materials and our previous studies (*57*, *58*).

For a pure solvent, the permeate flux depends only on the applied pressure and *f*_*f*−*m*_ as follows

$$\frac{dP}{dx}=-RTf_{f-m}v_{f}$$ (4)

where *P* is the applied pressure. Note that the solvent permeance (i.e., *A* in Eq. 2) is inversely proportional to *f*_*f*−*m*_

$$A=\frac{1}{RTf_{f-m}L_{m}}$$ (5)

where *L_m_* is the membrane active layer thickness. We calculated the *f*_*f*−*m*_ coefficient for the polyamide membrane for the various solvents on the basis of the experimentally measured *A* values (fig. S13). The calculation of *f*_*f*−*m*_ for the cellulose triacetate membrane is not possible because the membrane is asymmetric without a well-defined selective layer thickness as the TFC polyamide membrane. We also calculated the *f*_*f*−*m*_ for water and methanol on the basis of the NEMD results for the polyamide membrane, which agree notably well with those obtained from the solvent permeation experiments (fig. S13).

For electrolyte solutions, the salt transport equation must be considered simultaneously with the force balance equation. Solving for *v_i_* using eq. S24 and substituting the expression for *v_i_* into Eq. 3 yields

$$-\frac{1}{RT}\frac{dP^{t}}{dx}=f_{f-m}v_{f}+\sum f_{i-f}c_{i}(1-K_{f,i})+\sum K_{f,i}\frac{{dc}_{i}}{dx}+\sum K_{f,i}c_{i}z_{i}\frac{d\varphi }{dx}$$ (6)

where *K*_*f*,*i*_ is the frictional factor of ion *i* due to the interactions of ion *i* with the membrane and water (eq. S25), *z_i_* is the ion valence, and ϕ is the electrical potential. *K*_*f*,*i*_ ranges from 0 to 1, where *K*_*f*,*i*_ = 0 and *K*_*f*,*i*_ = 1 indicate infinite friction and no friction between solutes and the membrane, respectively.

For neutral solutes, the electrical potential term in Eq. 6 vanishes. In addition, assuming that there is no friction between the neutral solutes and membrane matrix (i.e., *K*_*f*,*i*_ = 1), we can write the water velocity as (derivation in the Supplementary Materials)

$$v_{f}=A(\text{Δ}P-\sigma \text{Δ}\pi )$$ (7)

where σ is the reflection coefficient (defined in the Supplementary Materials) and Δ*P* and Δπ are the hydrostatic and osmotic pressure differences across the membrane, respectively. We note that Eq. 7 is the same as the well-known Spiegler-Kedem-Katchalsky (SKK) model that was developed on the basis of irreversible thermodynamics for a two-component system (i.e., water and salt), without accounting for the interactions between the species and membrane matrix (*60*, *61*). Therefore, *K*_*f*,*i*_ = 1 is explicitly assumed by the SKK model. However, unlike the SKK model, which relies on phenomenological parameters, the SF model provides mechanistic insights about the transport of water in the membrane.

Assuming *K*_*f*,*i*_ = 1 and including the effect of membrane charge and the electrical potential gradient—typical for electrolyte solutions and charged membrane—an expression for the water permeate flux can be obtained

$$v_{f}=-\frac{1}{f_{f-m}RT}\frac{dP}{dx}+\frac{\omega X}{f_{f-m}}\frac{d\varphi }{dx}$$ (8)

where ω is the sign of the membrane charge (i.e., −1 for a negatively charged membrane and +1 for a positively charged membrane) and *X* is the membrane charge density (i.e., ∣*X*∣ is equal to the concentration difference between cations and anions within the membrane). Equation 8 indicates that the water permeance could be influenced by the electrical potential term (second term on the right-hand side of the equation), which is also influenced by salt concentration. However, previous studies did not show a dependence of water permeance of salt concentration (*57*) because of the relatively low membrane charge and electrical potential gradient for both the polyamide and cellulose triacetate membranes. Notably, the polyamide membrane has a relatively low charge (~100 mM) (*62*, *63*), and the cellulose triacetate membrane is nearly neutral (*64*). In the following discussion, we demonstrate the robustness and accuracy of the SF model in predicting the influence of electrical potential on water permeance by conducting experiments with highly charged ion exchange membranes.

**Fig. 4.**
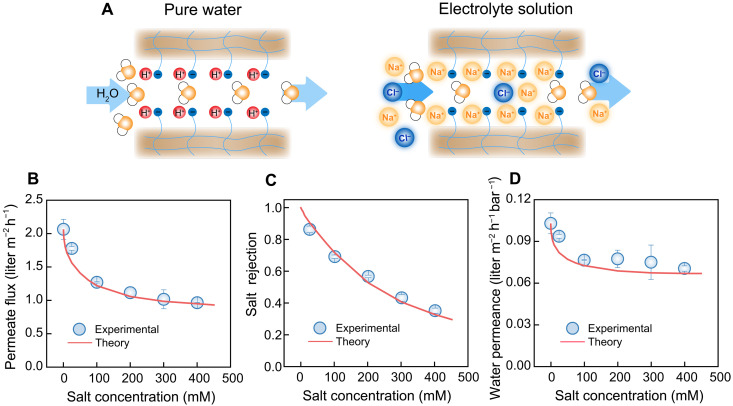
Effect of fixed membrane charge on water permeance. (**A**) Schematic illustration of the transport of pure water (left) and electrolyte solution (right) through charged membrane nanopores. (**B**) Permeate flux, (**C**) salt rejection, and (**D**) water permeance as a function of salt concentration for a charged nanoporous membrane. During the experiments, salt (NaCl) concentration varied from 25 to 400 mM while the applied pressure was fixed at 20 bar to minimize membrane compaction. The open symbols are experimental results with error bars representing one standard deviation from duplicate experiments. The solid curves are generated by the solution-friction model at the same operating conditions (i.e., feed salt concentration and applied pressure). The parameters used in the model are summarized in the Supplementary Materials (table S5) (*57*, *58*, *65*–*67*).

Inside a highly charged ion exchange membrane, such as Nafion, the sulfonate functional groups fully dissociate (*65*). In the proximity of the negatively charged sulfonate functional groups, protons are present as counter ions in pure water, thus neutralizing the membrane fixed charge (Fig. 4A). As pure water permeates through a highly charged membrane under an applied pressure, no protons can permeate through the membrane because of charge neutrality. Thus, the membrane is effectively noncharged—protons and negatively charged groups have the same concentration. As a result, water permeation is primarily controlled by the friction between water molecules and the membrane matrix (*f*_*f*−*m*_).

When an electrolyte solution is in contact with the membrane, the cation concentration inside the membrane is much larger than the mobile anion concentration, with the difference being equal to the membrane charge density. The membrane charge density increases from zero (with pure water) and approaches a limiting constant value as the electrolyte solution concentration increases (*68*–*70*). We use a Langmuir-type equation to account for the change of membrane charge as a function of the bulk salt concentration (see the derivation in the Supplementary Materials) (*68*–*75*).

Upon the application of pressure to the feed solution, water and salt permeate through the membrane. Water permeate flux decreases as the feed salt concentration increases, resulting from the increased osmotic pressure in the feed (Fig. 4B), while salt rejection decreases with increasing salt concentration (Fig. 4C) due to Donnan equilibrium (*76*). Specifically, the Donnan potential, established at the interface between the membrane and electrolyte solution, decreases as the feed salt concentration increases. Accordingly, the repulsion of co-ions (i.e., the ions bearing the same charge as the membrane) by the membrane is weakened, resulting in an increase in salt flux and a decrease in salt rejection.

The water permeance can then be calculated via Eq. 6 for varying feed electrolyte concentrations (Fig. 4D). As shown, water permeance shows a dependence on the feed salt concentration, a phenomenon not observed for weakly charged desalination membranes (*57*). Across the membrane, electrical potential increases from the feed side to the permeate side because of the higher concentration of cations than anions inside the membrane. The increased electrical potential enhances the velocity of anions and retards the velocity of cations because equal fluxes for cations and anions are required to maintain electroneutrality of the permeate solution. According to Eq. 8, the positive electrical potential gradient (i.e., *d*ϕ/*dx*) decreases the water flux through the membranes, thus reducing the water permeance. The SF model fully captures the dependence of water permeance on salt concentrations, demonstrating the validity and reliability of the model in describing salt and water transport through polymeric membranes.

In addition, we examined the gradient of water content within the membrane by stacking four ion-exchange membranes into a dead-end cell. A pressure difference was applied to drive pure water through the stack of membranes. After achieving a steady permeate water flux, the cell was depressurized and the water content for each membrane was determined immediately. As shown in fig. S14, the water content remains constant (i.e., no clear gradient) as a function of position along the stacked membranes for the two types of ion-exchange membranes (i.e., Nafion 211 and Fumasep FKS 30). This observation is consistent with our NEMD simulations presented in previous sections, further demonstrating that the SD mechanism is also not valid for ion-exchange membranes.

## DISCUSSION

Despite the wide use of the SD model, its underlying assumption of concentration gradient–driven water transport had not been systematically examined prior to this study. Herein, we combined NEMD simulations, permeation experiments, and physical models to unravel the true driving force and mechanism for water transport in RO membranes. Our NEMD simulations demonstrate that the water concentration across the membrane is invariant, while the pressure decreases linearly along the direction of water permeation. The simulations further show that water travels in clusters through transiently connected pores or free volumes inside the polyamide membrane under the action of a pressure gradient. Collectively, these findings support a pore flow mechanism for water transport in RO membranes, not an SD mechanism.

Permeation experiments of various solvents through polyamide and cellulose triacetate RO membranes reveal the important role of pressure in the permeation of solvents through the membranes. Our experiments showed that an appreciable critical pressure must be overcome to induce solvent permeation when the solvent molecular size approaches the membrane pore size. Moreover, we found that solvent permeance depends on the solvent molecular size rather than the solvent solubility, in contrast with the SD model. Additional water permeation experiments with charged polymeric membranes further demonstrated that water flux can be well described by the SF model, where water transport is driven by a pressure gradient.

There is a substantial difference between the applied pressures used in our experiments (maximum, 60 bar) and the NEMD simulations (minimum, 300 bar). These high pressures in our NEMD simulations are applied to accelerate the simulations and gather enough statistics in our 70-ns simulations. With these high pressures, the flux-pressure relationship in Fig. 1 remains linear up to 1500 bar, showing no indication of deviation from linearity or an approach to a “ceiling flux” as predicted by the SD model for such high pressures. Deviation of solvent flux from linearity with increasing applied pressure was reported more than 50 years ago for the transport of organic solvents through highly swollen (up to 80%) rubber films (*13*, *44*). However, such deviation of solvent flux from linearity occurred at considerably lower pressures than those predicted by the SD model, which we attribute to compaction of the highly swollen films under increased pressure. In a recent study (*31*), it was argued that experimental observations of water flux (or water permeance) decline in high-pressure (up to 150 bar) RO of aqueous solutions (*77*) can be explained by the SD model. However, this study clearly showed that the observed water flux decline was caused by severe membrane compaction (*77*). Furthermore, the decrease in water permeance was observable already at pressures lower than 50 bar, whereas the SD model predicts the initiation of flux decline at pressures greater than ~400 bar (Fig. 1).

A model based on the poroelastic theory was recently developed, claiming to unify the pore-flow and SD models (*31*). The model, called the fluid-solid model, is a two-phase model where the solvent phase is treated as a Newtonian fluid and the membrane phase is treated as a homogeneous elastic polymer. The two phases are perfectly mixed as a binary composite, which is inappropriate to describe the actual heterogeneous structure of RO membranes. By coupling Fick’s second law of diffusion for the binary system and the Navier-Stokes equation for the solvent flow, the model results in quantitatively identical permeability for both the pore-flow and SD mechanisms. Naturally, solving the governing equations for the pore-flow and SD mechanisms together (i.e., coupled governing equations) enforces the model to make identical transport predictions for both mechanisms. This mathematical treatment results in describing the transport by pressure-driven flow at the pore level and that by concentration-driven transport at the membrane level. In principle, the two driving forces can have additive contributions to the total flux (i.e., *J*_total_ = *J*_PF_ + *J*_SD_); however, the driving forces cannot contribute to the same flux (i.e., *J*_total_ = *J*_PF_ = *J*_SD_) as unphysically enforced by the fluid-solid model. In this conflicting transport description, the true mechanism of water transport in RO membranes remains unclear as the model simply solves for the transport in two separate worlds, one where the transport is governed by chemical diffusion and one where the transport is driven by pressure. Notably, here, we demonstrated that the real-world transport mechanism is governed by pore flow.

As we discussed earlier, the SD model was derived on the basis of flawed assumptions, some of which were also discussed elsewhere (*57*, *78*). In this model, the water permeance is dependent on the product of the partition coefficient of water into the membrane and the diffusion coefficient of water within the membrane. Numerous efforts to prove this relationship focused on qualifying the partition (solubility) of water in the membrane, which involves measurement of the membrane water content (*44*, *79*). This paradigm was extended to organic solvent nanofiltration, with research focusing on correlating the membrane solvent flux to solvent solubility (*56*, *80*). These efforts, however, were not successful in improving our mechanistic understanding of water flow in RO membranes. Further, because RO membranes contain a network of pores and the water (or solvent) resides within these pores or free volume, we argue that filling these pores with water (or solvent) may not be considered as an equilibrium process described by solubility (partitioning), where the water molecules partition into another phase. Instead, this process can be simply considered as membrane pore hydration.

As suggested by our NEMD simulations, the pressure decreases linearly along the membrane thickness because of dissipation of energy caused by frictional forces acting on solvent molecules as they travel through the membrane pore network. Our analysis shows that transport of water and other solvents in RO membranes is well described by the SF model, which accounts for these frictional forces in the form of water-membrane and water–ion friction coefficients. These coefficients, which are used as fitting parameters in the model, cannot be determined directly from experiments. To overcome this limitation of the model, future MD simulation efforts should be directed toward computing these coefficients and characterizing their dependence on the membrane properties (e.g., membrane charge, chemistry, pore size, and structure). Such studies will enable construction of structure-transport relationships, paving the way for the design and development of high-performance membranes.

The revelation of the true mechanism for water and solvent transport in our study provides molecular-level guidelines for the design and fabrication of new membranes. Our study underscores the importance of the difference between the membrane pore size and the solvent molecular size in governing solvent permeance. In membrane-based organic solvent separations, tuning the membrane pore size to approach the size of undesirable permeants while using an appropriate solvent with a smaller molecular size could potentially achieve highly selective separations. However, the compatibility between the solvent and the membrane as well as the uniformity of membrane pores should also be considered. For desalination applications, fabricating a membrane with appropriate pore/free volume size and reduced friction toward water molecules could be a promising strategy to increase water permeance. Nevertheless, the impact of these design parameters on salt rejection should also be considered because of the trade-off between water permeance and water-salt selectivity.

## MATERIALS AND METHODS

### NEMD simulations

A schematic for a typical NEMD simulation is shown in Fig. 1A. The system has dimensions of 5.0, 5.0, and 19.0 nm in the *x*, *y,* and *z* directions, respectively. Two graphene pistons are placed at *z* = 0.0 and 19.0 nm. A 10.0-nm-thick polyamide membrane is inserted between the feed and permeate reservoirs. The coordinates of the membrane-reservoir interfaces are *z* = 6.0 and 16.0 nm. Periodic boundary conditions are only applied in the *x* and *y* directions. A few atoms shown as blue were randomly selected and fixed in space to prevent the membrane from drifting because of the solvent transport (*42*, *81*, *82*). Fixing these atoms mimics the mechanical support provided by the polysulfone support layer of the polyamide membrane in the experiments. Previous studies showed that fixing some of the polyamide atoms has no effect on solvent transport as the separation mechanism is governed by the active layer (*83*, *84*).

A parameterized polymer-consistent force field (PCFF) (*85*–*87*) was used to model the interactions between atoms of graphene, water (or methanol), and membranes. PCFF has been widely used to describe the various properties of compounds, such as elastic constants, cohesive energies, and mechanical properties. Nonbonded interactions including the Lennard-Jones (LJ) and Coulomb potentials are expressed as: $U_{\mathrm{nonbonded}}=C\frac{q_{i}q_{j}}{\varepsilon r_{ij}}+\varepsilon_{ij}\left[2{\left(\frac{\sigma_{ij}}{r_{ij}}\right)}^{9}-3{\left(\frac{\sigma_{ij}}{r_{ij}}\right)}^{6}\right]$, where *r_ij_* represents the interatomic distance between the *i*th atom and *j*th atom, σ*_ij_* defines the distance between two atoms having a minimum LJ potential energy, ε*_ij_* is the depth of LJ potential, ε is the dielectric constant, *C* is a conversion factor, and *q_i_* and *q_j_* are the charges of the *i*th atom and *j*th atom, respectively. The interatomic interactions between different types of atoms are described using the sixth-power combination rule: $\sigma_{ij}={\left(\sigma_{ii}^{6}+\sigma_{jj}^{6}\right)}^{\frac{1}{6}}/2^{\frac{1}{6}}$ and $\varepsilon_{ij}=\sqrt{\varepsilon_{i}\varepsilon_{j}}\;(2r_{ii}^{3}r_{jj}^{3})/(r_{ii}^{6}+r_{jj}^{6})$. Nonbonded interactions are truncated by using a cutoff of 1.0 nm. Particle-particle particle-mesh solver is used to calculate the long-range electrostatic interactions with a force tolerance of 10^−4^. All MD simulations are performed using the LAMMPS package (*32*). The integration method is based on the velocity-Verlet algorithm, and the time step is 1.0 fs. First, energy minimization is carried out. Then, a standard atmosphere (1 bar) is imposed on the two graphene pistons to perform equilibrium MD (EMD) simulations for over 60.0 ns under NVT ensemble where temperature is maintained at 300 K.

The final configurations from the EMD simulations are used as the starting point for the NEMD simulations. The driving force is developed by applying a pressure difference (Δ*P* = 300, 600, 900, 1200, or 1500 bar) between the two graphene sheets. Each NEMD simulation is performed for more than 70 ns under the NVT ensemble. Initial atomic velocities are generated on the basis of a Gaussian distribution. The system temperature is maintained at 300 K using the Nosé-Hoover thermostat (*88*, *89*).

### Chemicals and materials

All solvents used in this study were American Chemical Society (ACS) grade. Methanol, ethanol, formamide, iso-propanol, *n*-propanol, 2-butanol, *n*-butanol, and n-hexane were purchased from Sigma-Aldrich. Deionized (DI) water was obtained from a Milli-Q ultrapure water purification system (Millipore, Billerica, MA). NaCl electrolyte solutions were made by dissolving ACS-grade sodium chloride (Thermo Fisher Scientific, Pittsburgh, PA) in DI water.

Commercial polyamide TFC membrane (SW30XLE, Dow Chemical Company, Midland, MI), cellulose triacetate membrane (FTSH2O, Fluid Technology Solutions, Albany, OR), Nafion membrane (Nafion 211, Fuel Cell Store, College Station, TX), and Fumasep ion exchange membrane (FKS-30, Fuel Cell Store, College Station, TX) were used in our experiments. Membranes were received as flat sheets and were thoroughly rinsed with DI water. Following the rinsing, all membranes were stored in DI water at 4°C before use.

### Permeation experiments

The permeation of solvents and NaCl electrolyte solutions were performed with a high-pressure dead-end cell (HP4750, Sterlitech, Auburn, WA). The cell accommodated a circular membrane sample with an active area of 12.56 cm^2^, which was supported by a stainless steel porous frit. The pressure was supplied by a high-pressure nitrogen gas tank (Airgas USA, Radnor, PA).

During the solvent permeation experiments through polyamide and cellulose triacetate membranes, the cell was placed on an analytical balance (Denver Instrument, Bohemia, NY), and the mass change (∆*m*) was recorded at a fixed time interval (∆*t*). Before applying pressure to the cell, the solvent was equilibrated with the membrane for ~2 hours. The solvent flux (*J*) was calculated on the basis of the slope (*m*) of a plot of ∆m vs ∆*t* using

$$J=\frac{m}{M_{w}A_{m}}$$ (9)

where *A_m_* is the active membrane area (12.56 cm^2^) and *M_w_* is the solvent molecular weight. Each permeation experiment was conducted for at least 1 hour.

For permeation experiments with pure water and electrolyte (NaCl) solutions through the Nafion membrane, the cell was placed on a stir plate with a magnetic stirrer inside the cell. A stirring rate of 1000 rpm was set to minimize the concentration polarization during the salt rejection experiments. The permeate water flux through the Nafion membrane ($J_{w}^{N}$) was calculated from the cumulative permeate volume (∆*V*) collected in a glass beaker via

$$J_{w}^{N}=\frac{\text{Δ}V}{A_{m}t}$$ (10)

where *t* is the permeation duration (typically 1 hour). Over the testing period, the concentration change of the salt solution in the cell was negligible, as the permeate water volume did not exceed 3 ml, while the initial volume of the salt solution was 250 ml. The solution conductivity was measured with a calibrated conductivity probe (Oakton CON110, Oakton Instruments, Vernon Hills, IL). The conductivity was then converted to concentration using a calibration curve. For both experimental and modeling data, the observed salt rejection (*R_j_*) was calculated using the feed and permeate concentrations by

$$R_{j}=1-\frac{c_{p}}{c_{f}}$$ (11)

where *c_f_* and *c_p_* are the salt concentration in the feed solution and permeate solution, respectively. The water permeance through the Nafion membrane ($A_{w}^{N}$) was calculated by normalizing the water flux ($J_{w}^{N}$) by the driving force across the membrane:

$$A_{w}^{N}=\frac{J_{w}^{N}}{\text{Δ}P-2RT(c_{w}-c_{p})}$$ (12)

where ∆*P* is the applied pressure and *c_w_* is the salt concentration near the membrane surface. The latter is nearly the same as *c_f_* due to negligible concentration polarization, resulting from rigorous mixing, very low permeate water flux (typically less than 2.0 liter m^−2^ h^−1^), and relatively low salt rejection. Details on the calculation of concentration polarization are provided in the Supplementary Materials.

### Water content measurement

Four identical membranes were mounted in a high-pressure dead-end with DI water as the feed. A pressure of 40 and 60 bar was applied for the Nafion and FKS-30 membranes, respectively. The system was set to equilibrate with the pressure for more than 4 hours. The testing cell was then disassembled, and the weights of the wet membranes (*W*_wet_) were immediately measured. After the measurements, the membranes were dried in a vacuum oven at ~65°C and less than −70-cmHg pressure overnight. Afterward, the dry weights of the membranes (*W*_dry_) were measured. The water content ratio of the membranes was calculated as (*W*_wet_ − *W*_dry_)/*W*_dry_. The tests were repeated three times with the positions of the membrane sample coupons shuffled.
