# Supplementary material for: Water transport in reverse osmosis membranes is governed by pore flow, not a solution-diffusion mechanism
Source: Sci Adv. 2023 Apr 14;9(15):eadf8488. doi: 10.1126/sciadv.adf8488 (PMC10104469; doi:10.1126/sciadv.adf8488)
Supplement: Supplementary file 1 — Supplementary Text Figs. S1 to S14 Tables S1 to S5 [file sciadv.adf8488_sm.pdf]

Science Advances
AAAS

Supplementary Materials for

Water transport in reverse osmosis membranes is governed by pore flow, not a solution-diffusion mechanism

Li Wang *et al.*

Corresponding author: Menachem Elimelech, menachem.elimelech@yale.edu

Sci. Adv. **9**, eadf8488 (2023)
DOI: 10.1126/sciadv.adf8488

This PDF file includes:

Supplementary Text
Figs. S1 to S14
Tables S1 to S5

Supplementary Text

Atomistic models for polyamide membranes fabricated by the interfacial polymerization method

In the experimental fabrication of polyamide membranes by the interfacial polymerization (IP) method *(33, 34, 35)*, trimesoyl chloride (TMC) and a diamine, such as m-phenylene diamine (MPD), first dissolve into an aqueous solvent and an organic solvent, respectively. The two solvents are immiscible. Next, a polysulfone film-based microporous substrate is inserted into the MPD solution to allow MPD monomers diffuse into the TMC solution through the supporting film. With the MPD monomers diffusing into the organic solution, the polymerization reaction occurs at the aqueous/organic solvent interface *(36)*. As the reaction proceeds, a polyamide layer is formed. This layer eventually prevents the MPD monomers from diffusing into the TMC solution, leading to a self-limiting process with a varying density of polyamide cross-linking.

To model the experimental IP process for building a 10.0 nm-thick polyamide membrane, 690 MPD and 460 TMC monomers are considered in this study. These MPD and TMC monomers provide up to 1,380 possible reaction sites. All monomers are packed into two separate 3D-periodic cells. The dimensions of each cell are 5.0 nm × 5.0 nm × 5.0 nm. Similar to the experimental IP process, the cross-linking reactions take place at the interface between the two cells as shown in fig. S1.

Cross-linking reaction process for generating an atomistic model of polyamide membranes

In order to develop the atomistic model of polyamide membrane by the IP method, a multi-step cross-linking process was implemented *(37)*. As shown in fig. S1B, an initial configuration was generated by performing a geometry optimization followed by 20 cycles of annealing. Periodic boundary conditions (PBC) were only imposed in the *x* and *y* direction. A vacuum space was inserted in the *z* direction of the simulation box. Next, the polymerization simulation was carried out in the canonical ensemble (NVT). During the simulation, cross-linking reactions occur when the bonding sites were within an initial cutoff distance of 4.5 Å. Virtual elastic springs were created between acyl carbon (OH-C=O-) atoms of TMC and amino nitrogen (-NH_2) atoms of MPD within the cutoff distance. The reactive atoms were pulled together using the virtual elastic springs. When the distance between the given reactive atoms was less than 1.5 Å, the cross-linking bond (C-N) was developed. The cross-linked polyamide configuration was further optimized for over 500 ps

under the isothermal–isobaric ensemble (NPT). The cross-linking reaction simulations are performed again with a cutoff of 0.5 Å until the reaction process stops by itself.

During the cross-linking reaction process, the degree of cross-linking (DC) was calculated at each reaction step using $DC\ (\%) = 100 \times \frac{\text{number of the developed C−N bonds}}{\text{number of the potential C−N bonds}}$. The final DC was obtained when the cross-linking reaction process stops by itself. Real-time partial charges were renovated through force-field assignment. A configuration optimization and 60 cycles of annealing were further conducted for the generated PA membrane. The final PA membrane model is shown in fig. S1C. The cross-linking polymerization simulation was performed in the absence of explicit solvents. Excluding the explicit solvents should not affect the polymerization process since solvents can eventually diffuse into the solution during the process *(38)*. The explicit solvents were excluded as their interactions with both membranes and solvents result in a retarded polymerization process and high computational cost.

Membrane solvation

We computationally built a model of a polyamide membrane with a 3:2 MPD/TMC ratio and an approximate thickness of 10.0 nm. The *DC* was determined by the self-limiting nature of the IP method. Water and methanol are considered as our solvents. The atomistic model of polyamide was employed to perform non-equilibrium molecular (NEMD) simulations of the RO process. Each simulation box consists of a 10 nm-thick polyamide membrane, solvent molecules in the feed and permeate reservoirs, and two piston graphene sheets. For each system, we performed three simulations with different initial configurations to obtain the error bars associated with our calculations.

To match the experimental water or methanol content (23.0 wt% for water and 0.792 g cm^{-3} for methanol) within the polyamide membrane, hydration MD simulations, where two 5.0 nm by 5.0 nm graphene sheets were placed at both ends to maintain the pressure of reservoirs at the standard atmosphere (0.1 MPa), were performed to achieve the target water (or methanol) content. As shown in Fig. 1A, water molecules (or methanol) penetrated the available free space until we reached the target water (or methanol) content. The assembled system was employed to carry out an equilibrium simulation under the NVT ensemble. The equilibrated system was then employed to perform the NEMD simulations.

Analyzing water transport in our NEMD simulations

a) Solvent flux and membrane permeance. We calculated the number of permeated water or methanol molecules as a function of simulation time for different applied pressures. Permeance or flux was obtained from the slope of the curve.

b) Number of solvent molecules along the z direction. The last 1 ns of the simulation was considered. Each configuration during the last 1 ns was divided into several slabs with a spacing of 0.5 nm along the membrane thickness (*z*-direction). The number of solvent molecules (water or methanol) was counted in each slab. The final reported number of solvent molecules was a result of averaging over the last 1 ns.

c) Density profiles. In the slabs discussed in (**b**), the numbers of molecules for the solvents and membrane are counted. For each slab, the local density was calculated by dividing the total mass by the slab volume. The final density profile was obtained by averaging over the last 1 ns.

d) Region partition criterion. We defined three regions within the system: the solvent, confined membrane, and transition region across the membrane. The bulk region is defined as the region where the solvent density is 0.99 g cm^{-3} for water or 0.792 g cm^{-3} for methanol. The confined membrane is the region with a constant low solvent density. The transition region is defined as between the edges of the bulk and confined membrane regions.

e) Membrane porosity. In the last 1 ns of the simulations, we calculated the total volume and geometric pore volume of the confined membrane using PoreBlazer *(39)*. The membrane porosity was expressed as the ratio of geometric pore volume to total volume. The final porosity is based on averaging over the last 1 ns of the simulation.

f) Pore size distributions (PSDs). PSDs were calculated by PoreBlazer. Studies have shown that the size of solvent molecules can be better characterized using their Coulombic diameter *(40)*. Polyamide membranes have several polar groups, including –COOH and $–NH_2$. Therefore, a probe diameter of 0.28 nm (for water) or 0.36 nm (for methanol) is used to evaluate the PSDs. These correspond to the Coulombic diameter of one water molecule or one methanol molecule. The last 10 frames of the NEMD simulations were chosen to compute the PSDs. The final PSDs were reported by averaging over the last 10 frames.

g) Pressure distribution along membrane thickness (z direction**).** After the system reached steady state under NEMD simulations, all atomic virial stress tensor elements of solvents and polyamide membrane were computed. The simulation system was divided into bins with a size of 0.5 nm along the z direction. The total pressure for each bin was obtained by summing up all the hydrostatic pressures.

h) Coordination number for water molecules. The coordination number of a reference atom (e.g., oxygen atom) in a water molecule refers to the total number of oxygen atoms or water molecules around the reference atom within a certain cutoff. After each system achieved a steady state under NEMD simulations, the coordinates of all water molecules were monitored during the last 1 ns. The number of oxygen atoms around the oxygen atom of each reference water molecule was counted where the cutoff distance was 0.5 nm. The probability distribution for the O-O coordination number was obtained by normalizing the coordination numbers by their frequency.

<u>Permeate flux based on the solution-diffusion model</u>

Details on the principles and derivation of the solution-diffusion (SD) model can be found elsewhere *(11, 12, 45)*. Here, we provide the key equation relating the solvent flux to the applied pressure. According to the SD model, the solvent permeate flux is driven by the concentration gradient of solvent across the membrane. Fick's law is therefore used to calculate the solvent flux (J_{SD}) following *(11, 12, 45)*

$$J_{SD} = \frac{D_m}{L_m}\left(c_{m,0} - c_{m,l}\right) \tag{S1}$$

where D_m is the diffusion coefficient of solvent inside the membrane, L_m is the membrane thickness, and $c_{m,0}$ and $c_{m,l}$ are solvent concentrations just inside the membrane on the feed and permeate sides, respectively.

The SD model assumes that the pressure inside the membrane remains constant and equal to the feed pressure, P_0, whereas a sudden drop of pressure, $P_0 - P_l$ (P_l, permeate pressure), occurs at the membrane-permeate interface. Based on this assumption and thermodynamic equilibria at the interfaces, the membrane concentrations in Eq. S1 are translated into the bulk solution concentrations *(12, 14, 45)*. The permeate flux can be, then, expressed as a function of the pressure difference ($P_o - P_l$):

$$J_{SD} = \frac{D_m K}{L_m}\left[c_0 - c_l \exp\left(\frac{-(P_o - P_l)\bar{v}}{RT}\right)\right] \quad \text{(S2)}$$

where K is the partitioning coefficient of the solvent into the membrane, c_0 and c_l are the bulk solvent concentrations in the feed and permeate sides, respectively, $(P_o - P_l) =$ is the pressure difference across the membrame which is equal to the applied pressure (atmospheric pressure at the permeate), $\bar{v}$ is the solvent molar volume, R is the gas constant, and T is the absolute temperature.

When the feed is a pure solvent, the feed and permeate concentrations, c_0 and c_l, are identical. The product of K and c_0 or c_l is the solvent concentration inside the membrane. We can then rewrite Eq. S2 as

$$J_{SD} = \frac{D_m c_{m,0}}{L_m}\left[1 - \exp\left(\frac{-(P_o - P_l)\bar{v}}{RT}\right)\right] \quad \text{(S3)}$$

The value of $c_{m,0}$ can be estimated as the fraction of the membrane that is filled with solvent. The maximum of $c_{m,0}$ is therefore reached when all the membrane voids are filled with the solvent. Eq. S3 suggests that J_{SD} approaches $D_m c_{m,0}/L_m$ as the pressure difference $(P_o - P_l)$ approaches a very large value (i.e., J_{SD} does not increase linearly with pressure difference). The synthesized membrane has a porosity of 23% based on the MD simulations. Hence, for water and methanol, $c_{m,0}$ is calculated as 1.278×10^4 mol m^{-3} and 5.685×10^3 mol m^{-3} (i.e., multiplying molar density by 23%), respectively. In addition, based on the mean squared displacement in our MD simulations at equilibrium state, the diffusion coefficients of water and methanol inside the membrane are computed as 2.48×10^{-10} m^2 s^{-1} and 1.078×10^{-10} m^2 s^{-1}, respectively. The thickness of the membrane (L_m) is taken as 10 nm. However, the effective length could be longer than this value due to the tortuosity of the membrane. As a result, J_{SD} is likely overestimated in our analysis. Even with this overestimation, J_{SD} based on the solution-diffusion model deviates significantly from the NEMD simulations as shown in Fig. 1C and fig. S5B.

Derivation of critical pressure and permeance

The derivation of the critical pressure for solvent permeation is adapted from a recent theoretical study that relates permeate flux to the energy barrier for nanofiltration membranes *(49)*. The free energy change of the solvent cluster to permeate through the membrane is contributed by the energy that resists the solvent permeation and the energy that drives the solvent permeation:

$$\Delta G = F_{res}y - \int F_{dr}dy \tag{S4}$$

where y is the permeation length, F_{res} is the resistance force, and F_{dr} is the driving force.

Assuming a cylindrical shape of the pore, we can estimate the driving force by Stokes' law *(50, 51)*:

$$F_{dr} = C_1\eta_s yu \tag{S5}$$

where C_1 is a proportionality factor, η_s is the solvent viscosity, and u is the solvent velocity through the pore. The velocity depends on the volumetric flux of the solvent:

$$u = \frac{4q}{\pi d^2} \tag{S6}$$

where q is the volumetric flux of the solvent, and d is the pore size.

The resistance force can be estimated by

$$F_{res} \approx \frac{k_BT}{d - d_0} \tag{S7}$$

where k_B is the Boltzmann constant, T is the absolute temperature, and d_0 is the molecular size of the solvent.

Substituting Eqs. S5 – S7 into Eq. S4 yields

$$\Delta G \approx \frac{k_BT}{d - d_0}y - \int_0^y \frac{4q\eta_s y}{\pi d^2}dy = -\frac{2}{\pi}C_1\eta q\left(\frac{y}{d}\right)^2 + \frac{y}{d - d_0}k_BT \tag{S8}$$

The quadratic equation of ΔG indicates that the free energy increases and then decreases as a function of y. The maximum y_{max} defines the location of the energy barrier:

$$y_{max} = \frac{\pi}{4C_1}\frac{k_BT}{q\eta_s}\frac{d^2}{d - d_0} \tag{S9}$$

The maximum ΔG is

$$\Delta G_{max} = \frac{(2C_1 - 1)\pi}{8C_1^2}\frac{(k_BT)^2}{q\eta_s}\left(\frac{d}{d - d_0}\right)^2 \tag{S10}$$

Lumping the constants into C_2, Eq. S10 can be rewritten as

$$\frac{\Delta G_{max}}{k_BT} = C_2\left(\frac{k_BT}{q\eta_s}\right)\left(\frac{d}{d - d_0}\right)^2 \tag{S11}$$

We then define a critical energy barrier that should be independent of external forces *(51, 52)*:

$$\frac{\Delta G_c}{k_BT} = C_2\left(\frac{d}{d - d_0}\right)^2 \tag{S12}$$

To overcome the critical energy barrier, a critical volumetric flux should meet the following equations *(49)*:

$$q_c = \frac{\Delta G_c}{\eta_s} \tag{S13a}$$

$$q_c = C_2 \left(\frac{k_B T}{\eta_s}\right) \left(\frac{d}{d - d_0}\right)^2 \tag{S13b}$$

By comparing Eqs. S11 and S13b, we can find that the energy barrier ΔG_{max} is related to the critical flux via

$$\Delta G_{max} = k_B T \left(\frac{q_c}{q}\right) = k_B T \left(\frac{P_c}{P}\right) \tag{S14}$$

where P_c and P are the critical pressure and the applied pressure, respectively.

The molecular flux through nanopores can be expressed as a function of the energy barrier:

$$J = k_r \exp\left(-\frac{\Delta G_{max}}{k_B T}\right) \tag{S15}$$

where k_r is the rate of incidence on the nanopores. When the viscosity is low (e.g., dilute solutions), k_r is proportional to $\frac{q}{q_c}$ *(53)*. We can rewrite Eq. S15 as

$$J = k \left(\frac{q}{q_c}\right) \exp\left(-\frac{q_c}{q}\right) = k \left(\frac{P}{P_c}\right) \exp\left(-\frac{P_c}{P}\right) \tag{S16}$$

with k being a proportionality factor that is inversely proportional to the solvent viscosity (η_s).

Rearranging Eq. S16, we obtain the permeance as

$$A = \frac{J}{\Delta P} = \frac{k}{P_c} \exp\left(-\frac{P_c}{P}\right) \tag{S17}$$

For solvents with molecular size (d_0) that is smaller than the membrane pore size (d), P_c is very small as $\frac{d}{d-d_0}$ becomes a significantly large number (Eq. S12) only when the molecular size and pore size are extremely close. Therefore, P_c can be easily overcome by a relatively small P. The solvent permeance can then be expressed as

$$A = \frac{J}{\Delta P} = \frac{k}{P_c} \tag{S18}$$

The above equation (eq. S18) indicates that A is constant for solvent permeation and hence J increases linearly with pressure.

In addition, P_c depends on the critical energy barrier, ΔG_c, through the following equation:

$$P_c = \frac{\Delta G_c}{v} \quad \text{(S19)}$$

where v is the effective volume of the solvent cluster that can be approximated as $\pi\left(\frac{d}{2}\right)^3$. Therefore, the critical pressure is related to the nanopore size and the solvent size:

$$P_c \propto \frac{k_B T}{d(d - d_0)^2} \quad \text{(S20)}$$

The permeance can then be expressed as

$$A \propto \frac{d(d - d_0)^2}{\eta_s} \quad \text{(S21)}$$

This equation (Eq. S21) is used to plot the curves in Figs. 3E and 3F.

Permeance within the solution-diffusion framework

According to the SD mechanism, solvent must "dissolve" into the membrane and then diffuse through the membrane under concentration gradient *(11, 12)*. The permeance is therefore related to the solvent solubility *(56)*:

$$A \propto \frac{\delta_{p,s}}{\eta_s d_0} \quad \text{(S22)}$$

where $\delta_{p,s}$ is the solubility parameter. This equation fails to predict the solvent permeances through the cellulose triacetate membrane as shown in fig. S12.

Derivation of the Solution-Friction Model

The solution-friction (SF) model is developed based on force balance on the species transporting through the membrane *(57, 58)*. For the permeation of water, the driving force (i.e., gradient of the total pressure) is balanced by the friction between the water molecules and pore walls as well as the friction between salt ions and water molecules. The force balance can be expressed as

$$\frac{dP^t}{dx} = -RTf_{f-m}v_f + RT\sum_i f_{i-f}c_i(v_i - v_f) \quad \text{(S23)}$$

where P^t is the total pressure (i.e., the applied pressure minus the osmotic pressure), v_i and v_f are velocities of ions and fluid, respectively, c_i is the ion concentration, f_{f-m} and f_{i-f} are the friction coefficients between the water and membrane and between ion and water, respectively, R is the gas constant, and T is the absolute temperature.

The ion velocity is contributed by convection caused by water velocity, ion diffusion, and ion electrical migration *(24)*:

$$v_i = K_{f,i}v_f - K_{f,i}\varepsilon D_i\left(\frac{d\ln c_i}{dx} + z_i\frac{d\varphi}{dx}\right) \tag{S24}$$

where $K_{f,i}$ is the frictional factor of ion i, ε is the effective porosity of the membrane, D_i is the diffusion coefficient of ion in bulk solution, z_i is the ion valence, and φ is the dimensionless electrical potential. Furthermore, $K_{f,i}$ is a function of f_{i-f} and the friction between the ion and membrane (f_{i-m}):

$$K_{f,i} = \frac{f_{i-f}}{f_{i-f} + f_{i-m}} \tag{S25}$$

This equation indicates that $K_{f,i}$ ranges from zero to 1, with zero indicating infinitely large friction and 1 meaning no friction between the salt ions and membrane.

Substituting Eq. S24 into Eq. S23 yields:

$$-\frac{1}{RT}\frac{dP^t}{dx} = f_{f-m}v_f + \sum f_{i-f}c_i\left(1 - K_{f,i}\right) + \sum K_{f,i}\frac{dc_i}{dx} + \sum K_{f,i}\,c_i z_i\frac{d\varphi}{dx} \tag{S26}$$

If we assume negligible friction between the ions with membrane (i.e., $K_{f,i} = 1$), Eq. S26 can be simplified as

$$-\frac{1}{RT}\frac{dP^t}{dx} = f_{f-m}v_f + \sum\frac{dc_i}{dx} + \sum c_i z_i\frac{d\varphi}{dx} \tag{S27}$$

In addition, for a membrane with a low membrane charge density, the electrical potential gradient is relatively small compared to the concentration gradient and thus can be neglected. Then, we obtain the following equation:

$$-\frac{1}{RT}\frac{dP^t}{dx} = f_{f-m}v_f + \sum\frac{dc_i}{dx} \tag{S28}$$

The total pressure depends on the applied hydrostatic pressure (P) and the osmotic pressure (π):

$$P^t = P - \pi \tag{S29a}$$

$$\pi = RTc_i \tag{S29b}$$

Substituting Eqs. S29a and S29b into Eq. S28 gives us the following equation:

$$\frac{d(P-\pi)}{dx} = -RTf_{f-m}v_f - \sum\frac{d\pi_m}{dx} \tag{S30}$$

where π_m is the osmotic pressure inside the membrane. A partitioning coefficient (Φ) can be used to relate π_m and the osmotic pressure in the solution outside the membrane (π_b):

$$\pi_m = \Phi\pi_b \tag{S31}$$

Then we have

$$\frac{d(P-\pi_b)}{dx} = -RTf_{f-m}v_f - \Phi\frac{d\pi_b}{dx} \tag{S32a}$$

$$v_f = -\frac{1}{RTf_{f-m}}\left(\frac{dP}{dx} - \frac{d\pi_b}{dx}\right) - \frac{\Phi}{RTf_{f-m}}\frac{d\pi_b}{dx} \tag{S32b}$$

We can further integrate Eq. S32b as across the membrane thickness, L_m:

$$\int v_f dx = -\frac{1}{RTf_{f-m}}\int d\,P + \frac{1}{RTf_{f-m}}\int d\pi_b - \frac{\Phi}{RTf_{f-m}}\int d\pi_b \tag{S33a}$$

$$v_f = \frac{1}{RTf_{f-m}L_m}\Delta P - \frac{1-\Phi}{RTf_{f-m}L_m}\Delta\pi_b \tag{S33b}$$

where ΔP is equal to the hydrostatic pressure applied to the feed (assuming the permeate side is unpressurized), and $\Delta\pi_b$ is the osmotic pressure difference across the membrane.

Let $\frac{1}{RTf_{f-m}L_m} = A$ and $1 - \Phi = \sigma$. We then obtain the permeate water velocity as

$$v_f = A(\Delta P - \sigma\Delta\pi_b) \tag{S34}$$

We note that Eq. S34 is identical to the well-known Spiegler-Kedem-Katchalsky (SKK) equation *(60, 61).*

On the other hand, if we include the electrical potential gradient, Eq. S27 can be simplified if we replace P^t with Eqs. S29a and S29b:

$$-\frac{1}{RT}\frac{dP}{dx} = f_{f-m}v_f + \sum c_i z_i \frac{d\varphi}{dx} \tag{S35}$$

Inside the membrane, the charge neutrality is maintained, which can be expressed as

$$\sum c_i z_i + \omega X = 0 \tag{S36}$$

Substituting Eq. S36 into Eq. S35 yields

$$\frac{1}{RT}\frac{dP}{dx} + f_{f-m}v_f - \omega X\frac{d\varphi}{dx} = 0 \tag{S37}$$

The above equation is used in our study to explain the effect of electrical potential and membrane charge on the permeate flux.

Modeling Salt and Water Transport in Ion Exchange Membranes

The SF model is a general theory for solute transport under the influence of pressure, electrical field, and solute concentration. The model has been applied to describe salt and water transport in electrodialysis *(58)*. In addition to the equations described previously, we must consider the zero net charge flux (i.e., no current through the membrane):

$$J_{ch} = \sum z_i J_i = 0 \tag{S38}$$

where J_{ch} is the net charge flux.

Furthermore, at the two interfaces of the membrane, salt ion partitioning into the membrane is governed by steric effect and Donnan equilibrium. The steric partitioning coefficient (Φ_{st}) can be estimated by

$$\Phi_{st} = (1 - \lambda_i)^2 \tag{S39}$$

where λ_i is the ratio between the ion Stokes size and pore size. Donnan equilibrium is also established and the partitioning coefficient (Φ_D) is calculated by

$$\Phi_D = \exp(-z_i \Delta\varphi_D) \tag{S40}$$

where $\Delta\varphi_D$ is the Donnan potential. Overall, the partitioning of ions into the membrane is quantified as

$$c_i = c_{b,i} \Phi_{st} \Phi_D \tag{S41}$$

where c_i is the ion concentration inside the membrane and $c_{b,i}$ is the ion concentration just outside the membrane.

The Donnan potential is a function of membrane charge density (X). As we discussed in the main text, the membrane charge density depends on the salt concentration outside the membrane. It has been shown that such dependence can be estimated with a Langmuir equation *(69-71)*:

$$|X| = \frac{|X_{max}|}{1 + K_L \exp(|\Delta\varphi_D|)} \tag{S42}$$

where X_{max} is the theoretical maximum charge density of the membrane and K_L is an empirical constant. Generally, as ion exchange membranes are usually highly charged for a wide range of salt concentrations *(68, 72)*, K_L is a very small number (Table S4) such that the membrane can reach X_{max} when in contact with a low salinity solution.

The theoretical maximum charge density is estimated using the following equation *(73)*:

$$|X_{max}| = \frac{IEC}{SD} \rho_w \tag{S43}$$

where IEC is the ion exchange capacity, SD is the swelling degree, and ρ_w is the water density. For the Nafion 211 membranes tested in our study, IEC and SD are reported to be 0.95–1.01 meq g^{-1} *(74)* and 0.16 *(75)*, respectively. Therefore, $|X_{max}|$ is calculated as 6.25 mol L^{-1} when the IEC is taken as 1.0 meq g^{-1}.

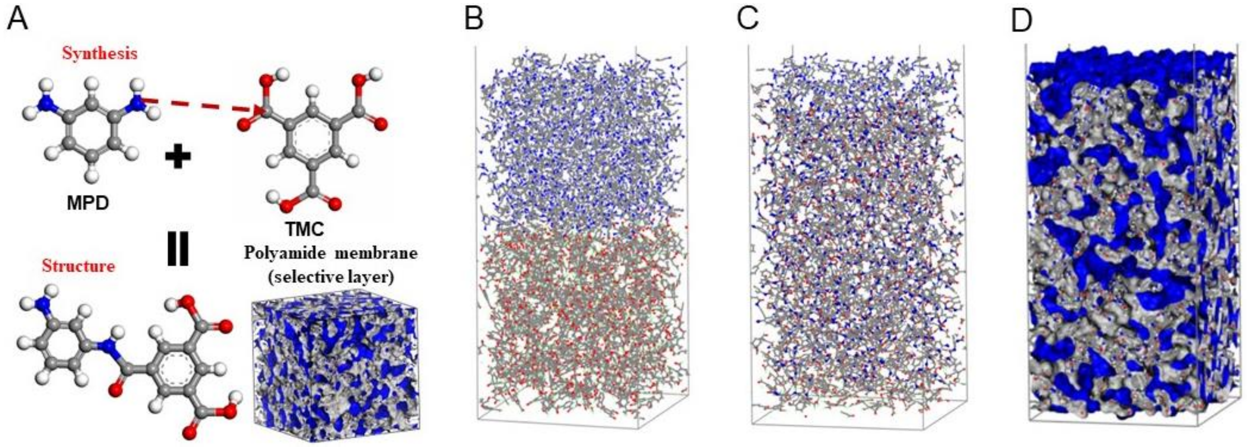

Fig. S1. Synthesis of a polyamide membrane by interfacial polymerization. (A) Chemical structures for two reacted monomers, i.e., TMC and MPD, and the cross-linking reaction forming the polyamide membrane. The interfacial polymerization (IP) process of a 10.0 nm-thick (vertical axis) atomistic polyamide membrane model is presented: (B) Snapshot of TMC and MPD monomers at the beginning of the interfacial polymerization process. The polymerization reaction is initiated at the interface of TMC (bottom) and MPD (top) solutions (solvents not shown). (C) Snapshot of polyamide membrane at the end of the interfacial polymerization process. The self-limiting mechanism of interfacial polymerization yields a cross-linking degree of 78.2% in the membrane. (D) Volume field of the fabricated polyamide membrane. The blue region represents the free volume of the membrane, and the gray region indicates the Connolly surface of the membrane.

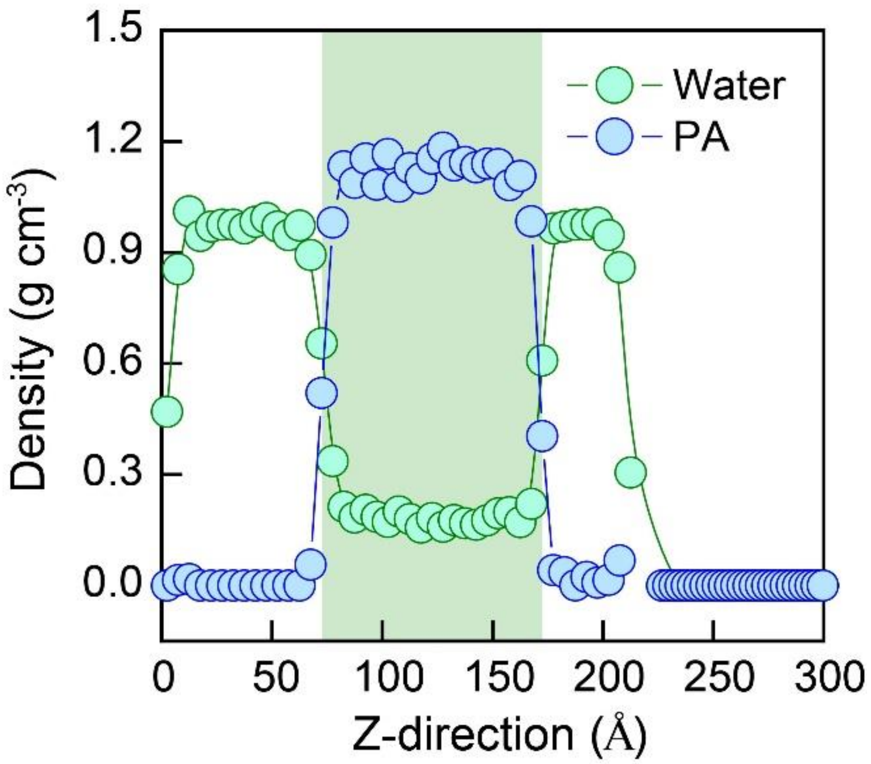

Fig. S2. Density profiles of water and polyamide along the z-direction at equilibrium. The mass density of polyamide (PA, blue circles) is zero outside the membrane (white area) and constant within the membrane region (shaded area). The mass density of water (green circles) is equal to the volumetric density of liquid water outside the membrane and drops to the level of membrane water content within the membrane.

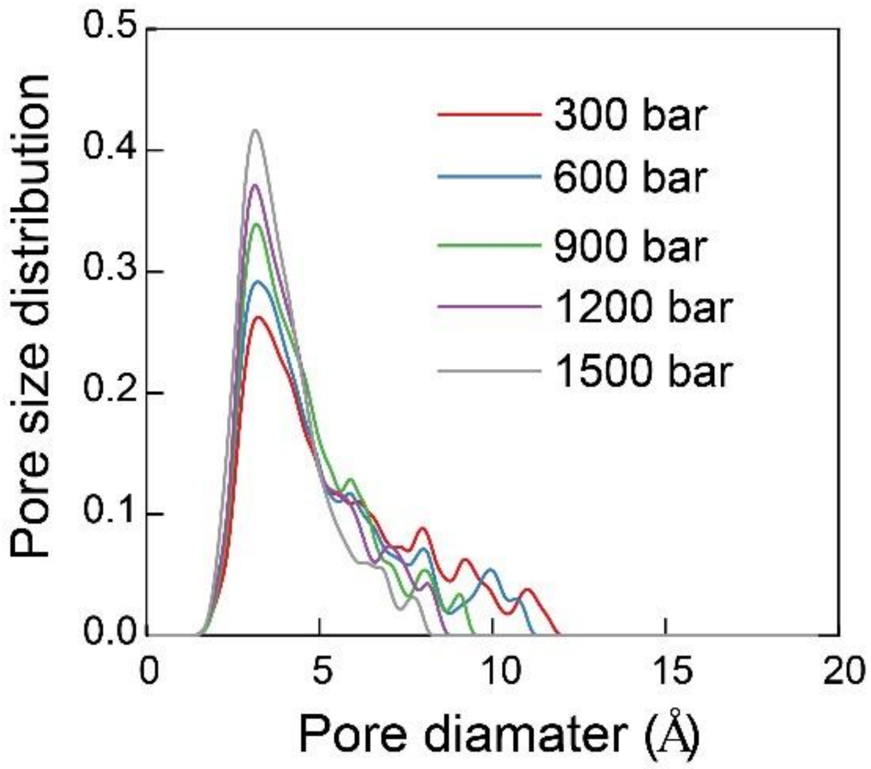

Fig. S3. Pore size distribution of synthesized polyamide membrane under varying applied pressure. The tested pressures are: 300, 600, 900, 1200, and 1500 bar.

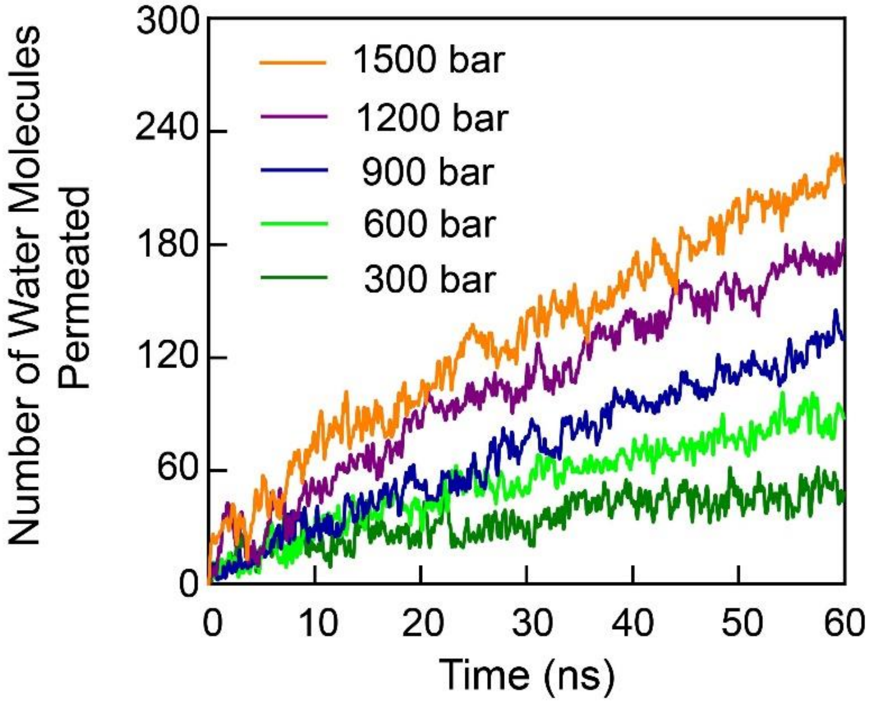

Fig. S4. Number of water molecules permeating through the polyamide membrane as a function of pressurized time at varying applied pressures. The numbers of water molecules permeated are recorded during the simulated tests under five pressure differences across the membrane (300, 600, 900, 1200, and 1500 bar). The results correspond to the water fluxes reported in Fig. 1C.

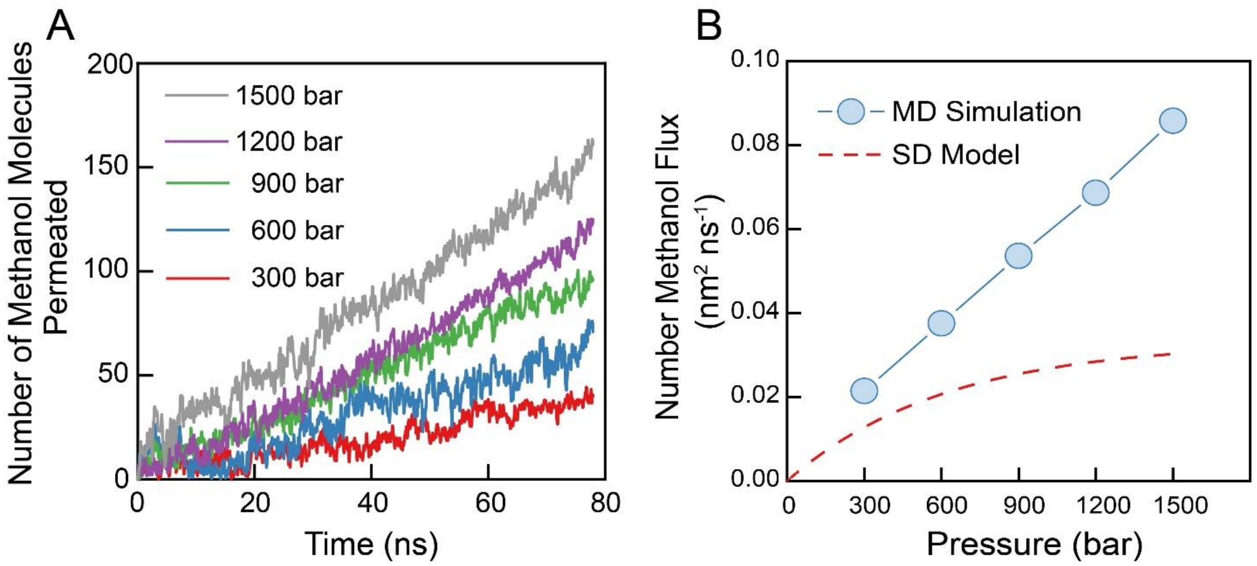

Fig. S5. Permeation of methanol through the synthesized polyamide membrane. (A) Number of methanol molecules permeating through the polyamide membrane as a function of pressurized time under five pressure differences across the membrane (300, 600, 900, 1200, and 1500 bar). (B) Methanol flux through the polyamide membrane as a function of applied pressure. The dashed red line is calculated based on the solution-diffusion model.

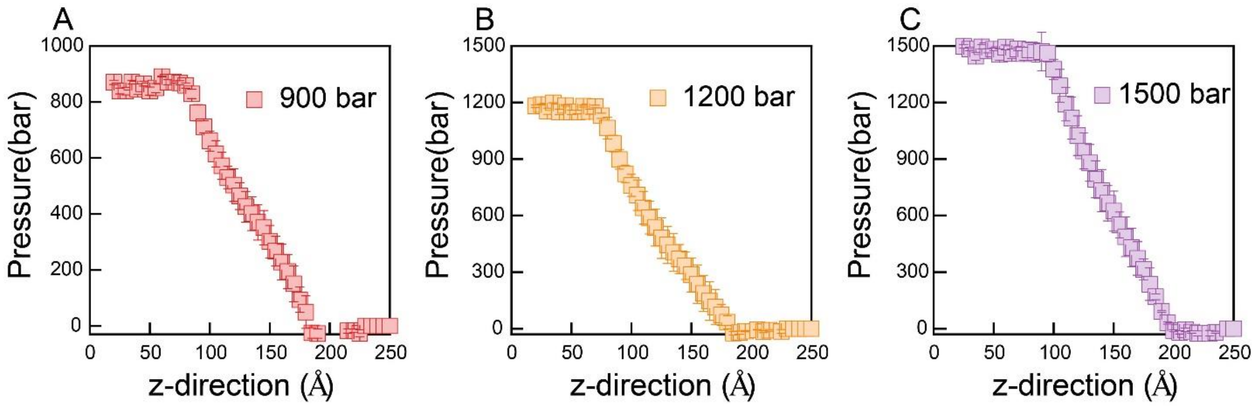

Fig. S6. Pressure distribution along z-direction through the polyamide membrane under three applied pressures: (A) 900 bar, (B) 1200 bar, and (C) 1500 bar. The pressure decreases linearly through the membrane from the feed side to permeate side.

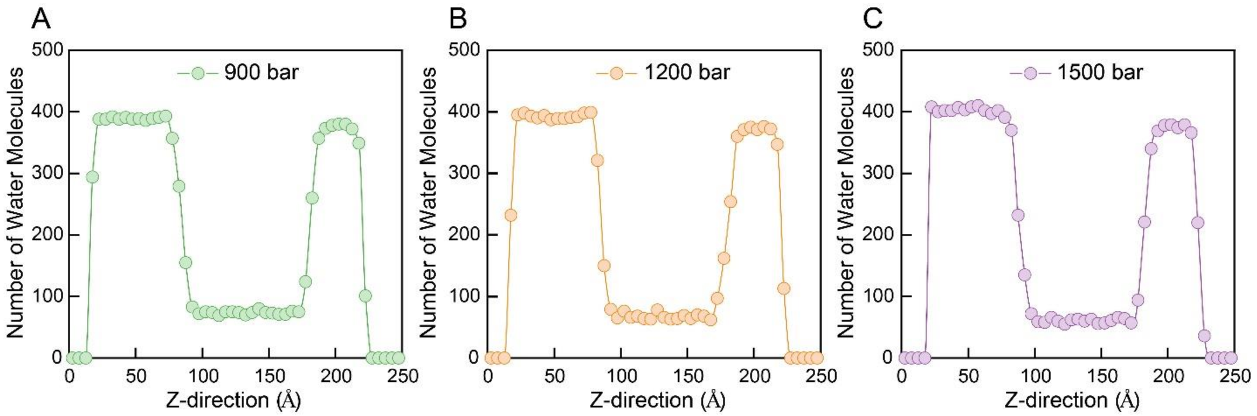

Fig. S7. Number of water molecules along z-direction through the polyamide membrane under varying pressures: (A) 900 bar, (B) 1200 bar, and (C) 1500 bar. The numbers of water molecules in the membrane are constant across the membranes. No water concentration gradient in the membranes is observed.

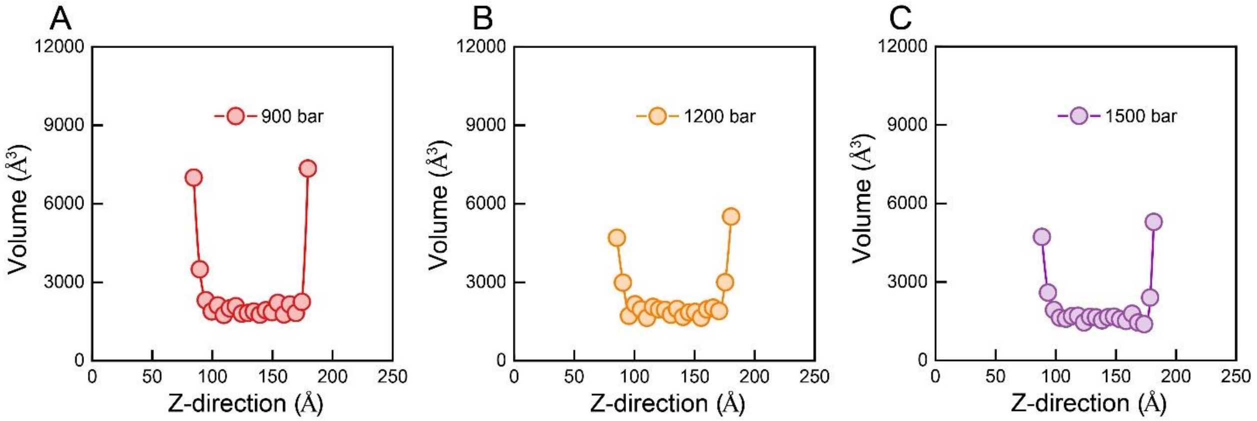

Fig. S8. Pore volume distribution along z-direction through the polyamide membrane under varying pressures: (A) 900 bar, (B) 1200 bar, and (C) 1500 bar. The pore volumes in the membrane are constant across the membrane despite the different applied pressures

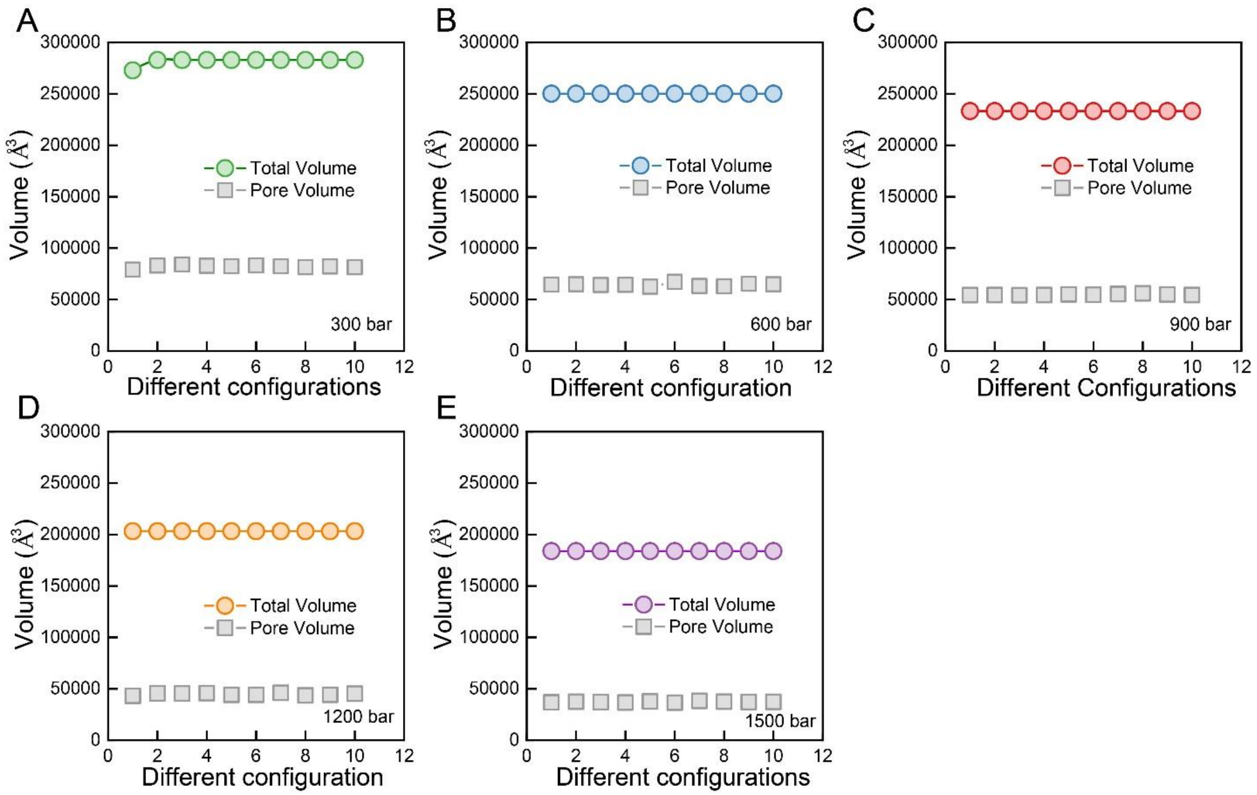

Fig. S9. Pore volume and total volume of the polyamide membrane under varying pressures: (A) 300 bar, (B) 600 bar, (C) 900 bar, (D) 1200 bar, and (E) 1500 bar. For each pressure condition, the volumes are calculated for ten configurations.

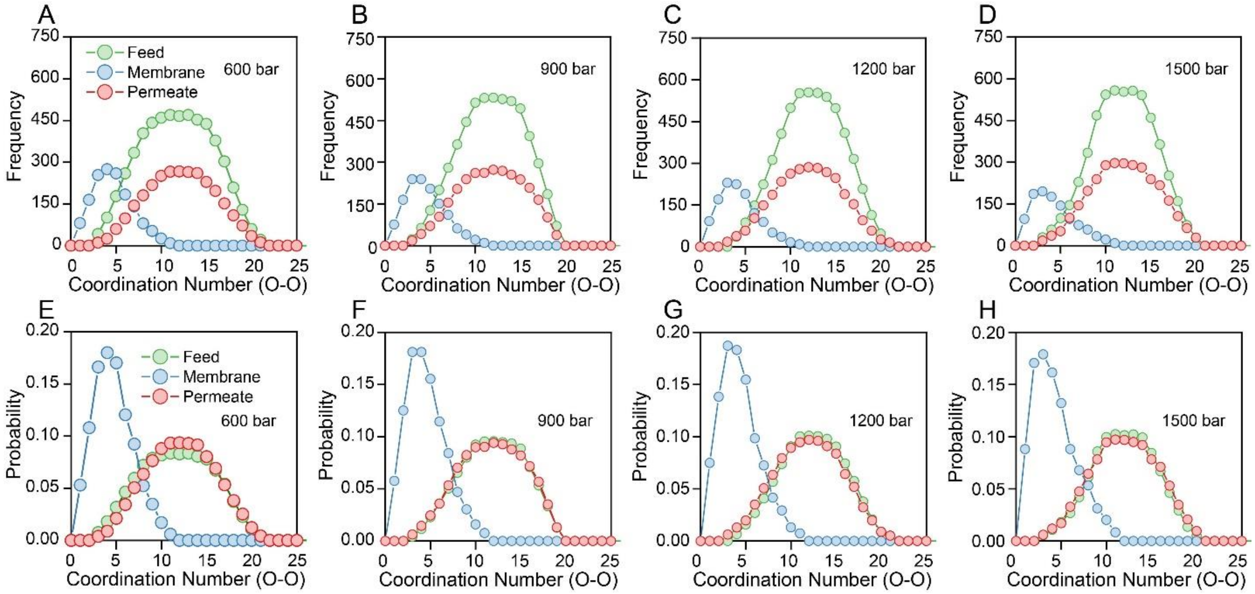

Fig. S10. Water clusters in the polyamide membrane. (A)-(D) Coordination number distribution of water molecules in the bulk feed, within the membrane, and in the permeate under four pressure differences across the membrane (600, 900, 1200, and 1500 bar). (E)-(H) Probability distribution of water molecules in the bulk feed, within the membrane, and in the permeate under four pressure differences across the membrane (600, 900, 1200, and 1500 bar)

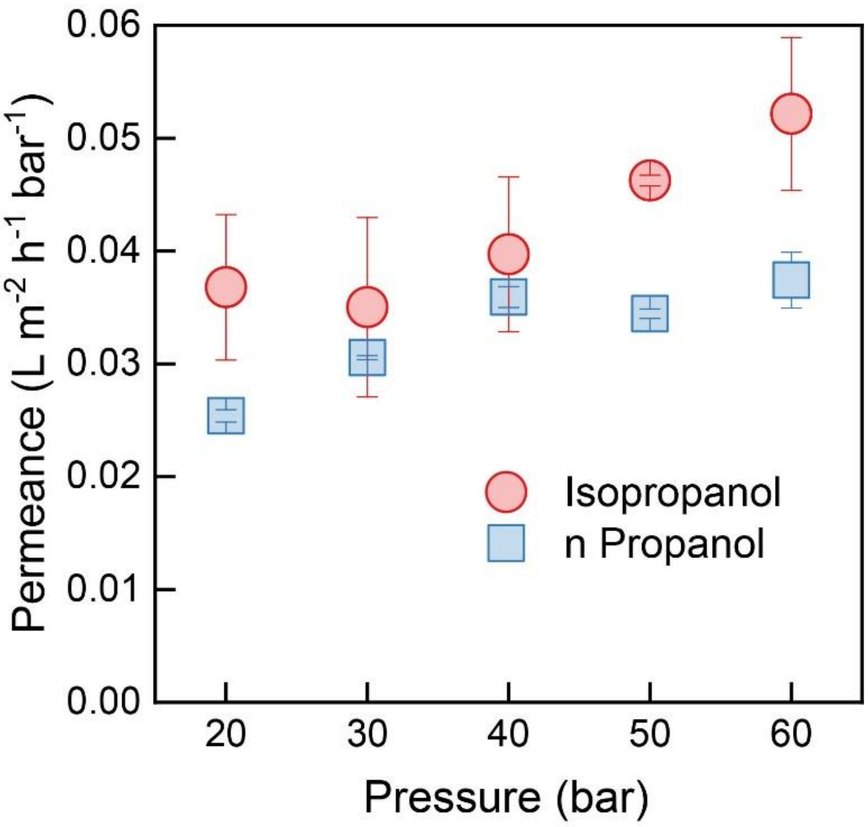

Fig. S11. Permeances of iso-propanol and n-propanol through the polyamide membrane as a function of applied pressure. The applied pressures are: 20, 30, 40, 50, and 60 bar.

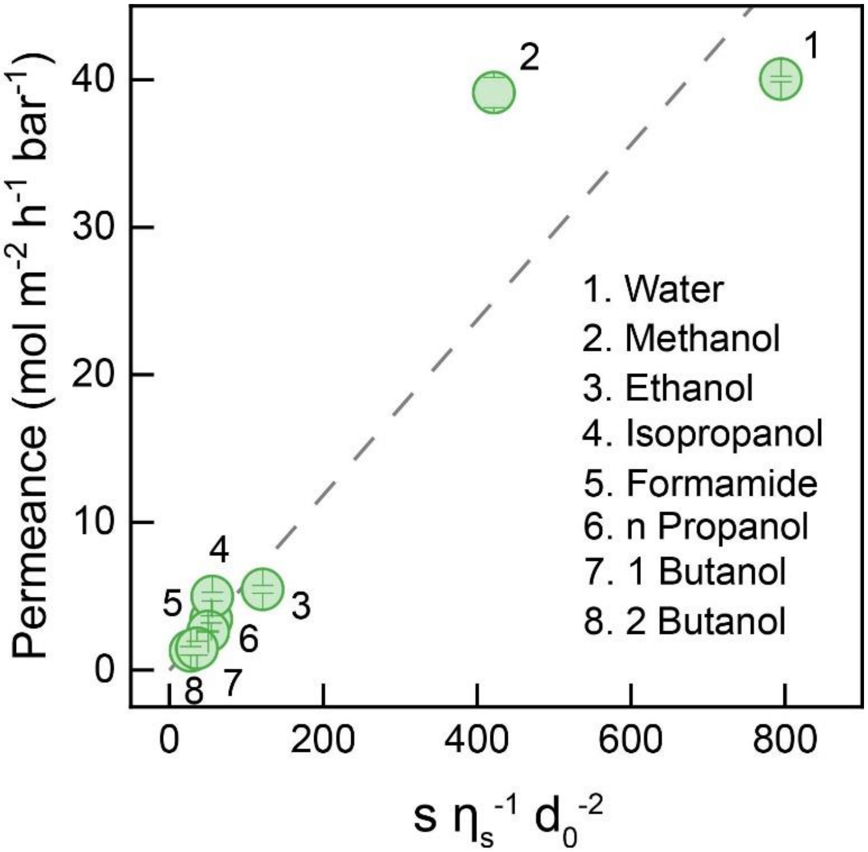

Fig. S12. Solvent permeance of cellulose triacetate membranes as a function of a model parameter combining viscosity (η_s), solvent size (d_0), and solubility parameter (s). The dashed line represents the linear relationship between the permeance and the combined model parameters. The open symbols are experimental results.

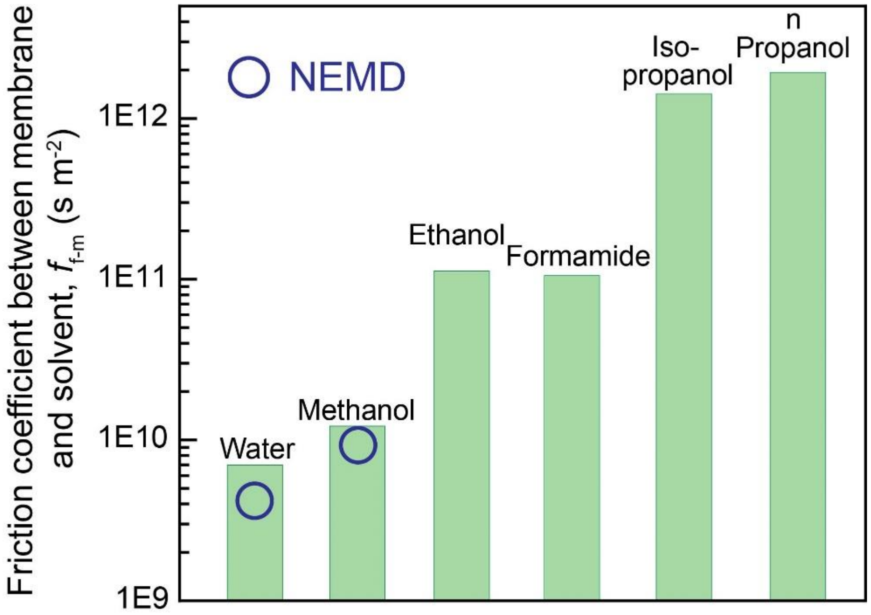

Fig. S13. Friction coefficient between the various solvents and the polyamide. The bars are experimental friction coefficients calculated from $\boldsymbol{f_{f-m} = 1/(RTAL_m)}$. The membrane thickness is assumed to be 150 nm. The open circles are friction coefficients obtained from NEMD results, based on the diffusion coefficients of water and methanol obtained from the corresponding mean-squared displacements.

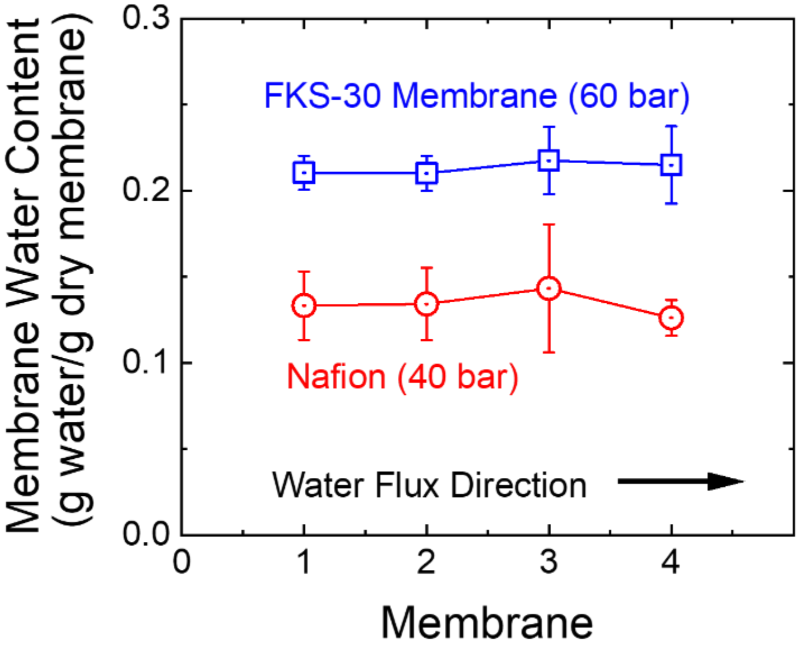

Fig. S14. Water content profile in a membrane stack consisting of four ion exchange membranes. Two types of ion exchange membranes, Nafion 211 (red) and Fumasep FKS-30 (blue), were tested. The water content was determined after achieving steady permeate water flux by applying 40 bar and 60 bar to Nafion and FKS-30, respectively. The membranes are numbered in the order from feed side to permeate side.

Table S1. Chemical composition and properties of the synthesized polyamide membrane

Atomic Composition					Degree of Cross-linking (%)	Density (g cm^{-3})
C (%)	N (%)	O (%)	COOH (%)	O/N (%)		
74.30	12.85	10.05	2.80	0.782	78.20	1.266

Table S2. Summary of solvent permeance for the polyamide and cellulose triacetate membranes

**Membrane**	**Solvent**	**Permeance or Slope (J vs P) (mol m^{-2} h^{-1} bar^{-1})**	**R^2**
Polyamide	Water	122.08	0.998
	Methanol	74.88	0.998
	Ethanol	9.02	0.995
	Formamide	10.27	0.996
Cellulose Triacetate	Water	40.17	0.999
	Methanol	37.13	0.994
	Ethanol	5.40	0.996
	Formamide	4.65	0.995
	n Propanol	2.81	0.998
	Isopropanol	2.85	0.994

Table S3. Critical pressures determined by fitting to Eq. 1

**Solvent**	**Critical Pressure P_c (bar)**	**Proportionality factor *k***	**R^2**
n-Propanol	13.3	8.3	0.991
Isopropanol	12.9	10.0	0.991

Table S4. Comparison between solvent solubility and solvent permeability

Solvent	Hansen solubility parameter ($MPa^{1/2}$)	Permeability ($mol\ m^{-2}\ h^{-1}\ bar^{-1}$)	
		**PA**	**CTA**
Water	47.8	138.65 ± 0.96	40.04 ± 0.18
Methanol	29.7	79.52 ± 3.98	39.13 ± 1.02
Ethanol	26.6	8.63 ± 0.74	5.46 ± 0.27
Formamide	36.7	9.17 ± 0.81	4.97 ± 0.29
Isopropanol	24.6	0.68 ± 0.09	3.43 ± 0.25
n-Propanol	23.6	0.5 ± 0.03	2.61 ± 0.04
2-Butanol	20.8	NA	1.29 ± 0.30
n-Butanol	23.1	NA	1.47 ± 0.48

Table S5. Parameters used in modeling the salt and water transport in Nafion

Parameter	Description	Value	Unit
D_{Na^+}	Sodium ion diffusion coefficient	1.33×10^{-9}	$m^2\ s^{-1}$
D_{Cl^-}	Chloride ion diffusion coefficient	2.03×10^{-9}	$m^2\ s^{-1}$
D	Salt (NaCl) diffusion coefficient	1.64×10^{-9}	$m^2\ s^{-1}$
d_{Na^+}	Sodium Stokes diameter[1]	0.368	nm
d_{Cl^-}	Chloride Stokes diameter[1]	0.242	nm
d_{pore}	Nafion membrane pore diameter[2]	2	nm
f_{f-m}	Frictional coefficient between water and the membrane pore matrix[3]	4.70×10^{13}	mol s m^{-5}
f_{Na-f}	Frictional factor between Na^+ and the fluid, calculated from $1/D_{Na^+}$.[4]	7.52×10^{10}	s m^{-2}
f_{Cl-f}	Frictional factor between Cl^- and the fluid, calculate from $1/D_{Cl^-}$.[4]	4.93×10^{9}	s m^{-2}
K_L	Constant relating the membrane charge density to $\Delta\varphi_D$ in Eq. S42 (fitting parameter in our model)	0.01	
K_f	Frictional factor defined by Eq. S25 (fitting parameter in our model)[5]	0.7	
L_m	Nafion 211 membrane thickness[6]	30	μm
R	Gas constant	8.314	J K^{-1} mol^{-1}
T	Absolute temperature	298	K
X_{max}	Membrane charge density[7]	6.25	M
ε	Membrane effective porosity[8]	0.16	

[1]Ref (66).
[2]Ref (65).
[3]f_{f-m} is calculated based on the pure water permeance (A) and $\frac{1}{\mathrm{RT}AL_m} = f_{f-m}$.
[4]Ref (57).
[5]The fitted K_f in our model is closed to the value reported in ref (58).
[6]provided by the manufacture specifications.
[7]X_{max} is calculated following Eq. S43.
[8]Ref (67).
